# The importance of standardization for biodiversity comparisons: A case study using autonomous reef monitoring structures (ARMS) and metabarcoding to measure cryptic diversity on Mo’orea coral reefs, French Polynesia

**DOI:** 10.1371/journal.pone.0175066

**Published:** 2017-04-21

**Authors:** Emma Ransome, Jonathan B. Geller, Molly Timmers, Matthieu Leray, Angka Mahardini, Andrianus Sembiring, Allen G. Collins, Christopher P. Meyer

**Affiliations:** 1 Department of Invertebrate Zoology, Smithsonian National Museum of Natural History, Washington DC, United States of America; 2 Moss Landing Marine Labs, Moss Landing, California, United States of America; 3 Joint Institute for Marine and Atmospheric Research, University of Hawai‘i at Mānoa, Honolulu, Hawaii, United States of America; 4 Ecosystem Sciences Division Pacific Islands Fisheries Science Center, National Oceanic and Atmospheric Administration, Honolulu, Hawaii, United States of America; 5 Smithsonian Tropical Research Institute, Panama, Republica de Panama; 6 Indonesian Biodiversity Research Center, Denpasar, Bali, Indonesia; 7 Department of Environmental Sciences, Udayana University, Denpasar, Bali, Indonesia; 8 National Systematics Laboratory, National Marine Fisheries Service (NMFS), Washington DC, United States of America; Academia Sinica, TAIWAN

## Abstract

The advancement of metabarcoding techniques, declining costs of high-throughput sequencing and development of systematic sampling devices, such as autonomous reef monitoring structures (ARMS), have provided the means to gather a vast amount of diversity data from cryptic marine communities. However, such increased capability could also lead to analytical challenges if the methods used to examine these communities across local and global scales are not standardized. Here we compare and assess the underlying biases of four ARMS field processing methods, preservation media, and current bioinformatic pipelines in evaluating diversity from cytochrome c oxidase I metabarcoding data. Illustrating the ability of ARMS-based metabarcoding to capture a wide spectrum of biodiversity, 3,372 OTUs and twenty-eight phyla, including 17 of 33 marine metazoan phyla, were detected from 3 ARMS (2.607 m^2^ area) collected on coral reefs in Mo’orea, French Polynesia. Significant differences were found between processing and preservation methods, demonstrating the need to standardize methods for biodiversity comparisons. We recommend the use of a standardized protocol (NOAA method) combined with DMSO preservation of tissues for sessile macroorganisms because it gave a more accurate representation of the underlying communities, is cost effective and removes chemical restrictions associated with sample transportation. We found that sequences identified at ≥ 97% similarity increased more than 7-fold (5.1% to 38.6%) using a geographically local barcode inventory, highlighting the importance of local species inventories. Phylogenetic approaches that assign higher taxonomic ranks accrued phylum identification errors (9.7%) due to sparse taxonomic coverage of the understudied cryptic coral reef community in public databases. However, a ≥ 85% sequence identity cut-off provided more accurate results (0.7% errors) and enabled phylum level identifications of 86.3% of the sequence reads. With over 1600 ARMS deployed, standardizing methods and improving databases are imperative to provide unprecedented global baseline assessments of understudied cryptic marine species in a rapidly changing world.

## Introduction

Coral reefs are among the most biologically diverse, complex, and economically valuable ecosystems on earth. They support fisheries, protect coastlines, provide jobs, and are a source of new medicines [1]. They harbor one quarter to one third of all marine species, with recent estimates suggesting that between 550,000–1,330,000 multi-cellular species inhabit coral reefs worldwide [2]; most of these are not yet named or described [1,3]. Coral reefs are also one of the most threatened habitats on the planet; they are highly susceptible to local impacts, such as sedimentation, pollution, and resource exploitation, and global impacts such as ocean warming and acidification [4–6]. An estimated loss of 19–61% coral cover has occurred in the past few decades, with further declines likely worldwide [7–9].

This alarming rate of loss creates an urgency to document and understand the spatial and temporal distribution of reef species, the processes regulating reef diversity, and the consequences of species loss for ecosystem function [10–13]. Despite the known importance of these ecosystems, the fate of most reef-associated metazoans has received little attention [14]. Previous efforts to assess reef diversity have focused on conspicuous groups that are easy to identify, such as corals, fish, and some mollusks [15–16], even though the majority of diversity lies in the multitude of small organisms living within the complex reef framework [17]. The challenges in extracting them, the lack of taxonomic expertise, and the sheer richness of these small and often rare species have made documentation difficult. However, with the development of standardized sampling devices and the exponential progress in metabarcoding techniques, our ability to examine this cryptic fauna is now possible [18– 20].

Autonomous Reef Monitoring Structures (ARMS) were first conceived in 2004 [21] and later deployed as part of the Census of Marine Life’s CReefs Program [17, 22] to sample coral reef diversity in a standardized fashion. ARMS are long-term collecting devices that mimic to some extent the structural complexity of coral reefs. They are composed of PVC plates constructed in alternating open and semi-closed layers forming a 9” x 9” x 9” tiered unit. Their use has expanded to exploit morphometric, barcoding, and metabarcoding techniques to assess diversity across a wide variety of taxonomic groups, habitats, and regions by many programs, e.g. coral reefs (e.g. NOAA’s Pacific Reef Assessment and Monitoring Program (RAMP; www.pifsc.noaa.gov/cred/survey_methods/arms/) and the Mo’orea Biocode Project (http://mooreabiocode.org/)) [19, 21–24], oyster reefs [18], and in coastal habitats in Europe (www.devotes-project.eu). With over 1600 ARMS currently deployed and the huge investment by a number of organizations, the standardization of processing methods is imperative if the subsequent data are to be comparable and used towards global assessments of diversity.

A field protocol was published by Leray and Knowlton [18] for the dismantling of ARMS and processing of samples with the goal of ensuring high quality DNA preservation of motile and sessile communities. However, with the rapid and global expansion of ARMS and variability in funding, chemical restrictions, and working conditions between projects, it is important to evaluate how variability in field protocols may affect the comparability of data between sites and independent studies. For example, three preservatives have been used or proposed to store samples for metabarcoding of the sessile and motile fauna (95% EtOH, 25% DMSO and RNAlater). Moreover, differences in filtration and washing procedures conducted to prevent the denaturation of DNA of the sessile fraction may bias the resulting community profiles.

To determine the impact of protocol variability on recovered community composition, we devised an experiment to assess processing methods and preservation techniques. We conducted this investigation in Mo’orea, French Polynesia, because a high-quality library of marine COI barcodes exists as a result of the Mo’orea Biocode Project [25], enabling us to compare metabarcoding results with a local, curated database against publically available databases. We contrast metabarcoding results with image analyses of the sessile community and discuss the feasibility of processing ARMS using one method, globally. We also investigate the limitations of current bioinformatic methods, given current reference databases, in retrieving accurate taxonomic identification for coral reef species and provide the first investigation into the cryptic coral reef ecosystem of Mo’orea, French Polynesia.

## Materials and methods

### ARMS deployment and collection

With permission from the French Polynesian Government under the Biocode Project, three ARMS were deployed subtidally (~14 m) on the fore-reef in Mo’orea, French Polynesia, in January 2012, and were left for two years to allow for colonization of benthic communities. Upon retrieval, a 106 μm nitex-lined crate was placed over the ARMS to limit the loss of motile organisms during transport. ARMS were kept submerged in 45 μm filtered aerated seawater during transportation and processed at the UC Berkeley Gump Field Station.

### Motile preservation experiment

Each ARMS unit was disassembled plate by plate following Leray & Knowlton [18]; however, the brushing of plates was avoided to prevent disturbance of microbial communities, allowing for microbial subsampling for other projects. Motile individuals were size fractioned through sterilized sieves into three portions: 106–500 μm, 500 μm– 2 mm, and > 2 mm. The two smaller fractions were rinsed with 45 μm filtered seawater (FSW) into a 45 μm net and divided into four equal subsamples for testing preservation biases. One subsample was preserved as a voucher, and the other three were preserved in 95% EtOH, 25% DMSO (0.25 M EDTA (pH 7.5), 25% DMSO, NaCl-saturated), and RNAlater (Life Technologies). All were stored at -20°C for one month before DNA extraction. The > 2 mm fraction was not assessed as it is consistently stored in 95% EtOH for barcoding and morphometric analysis.

### Sessile processing and preservation experiment

**Fig 1:**
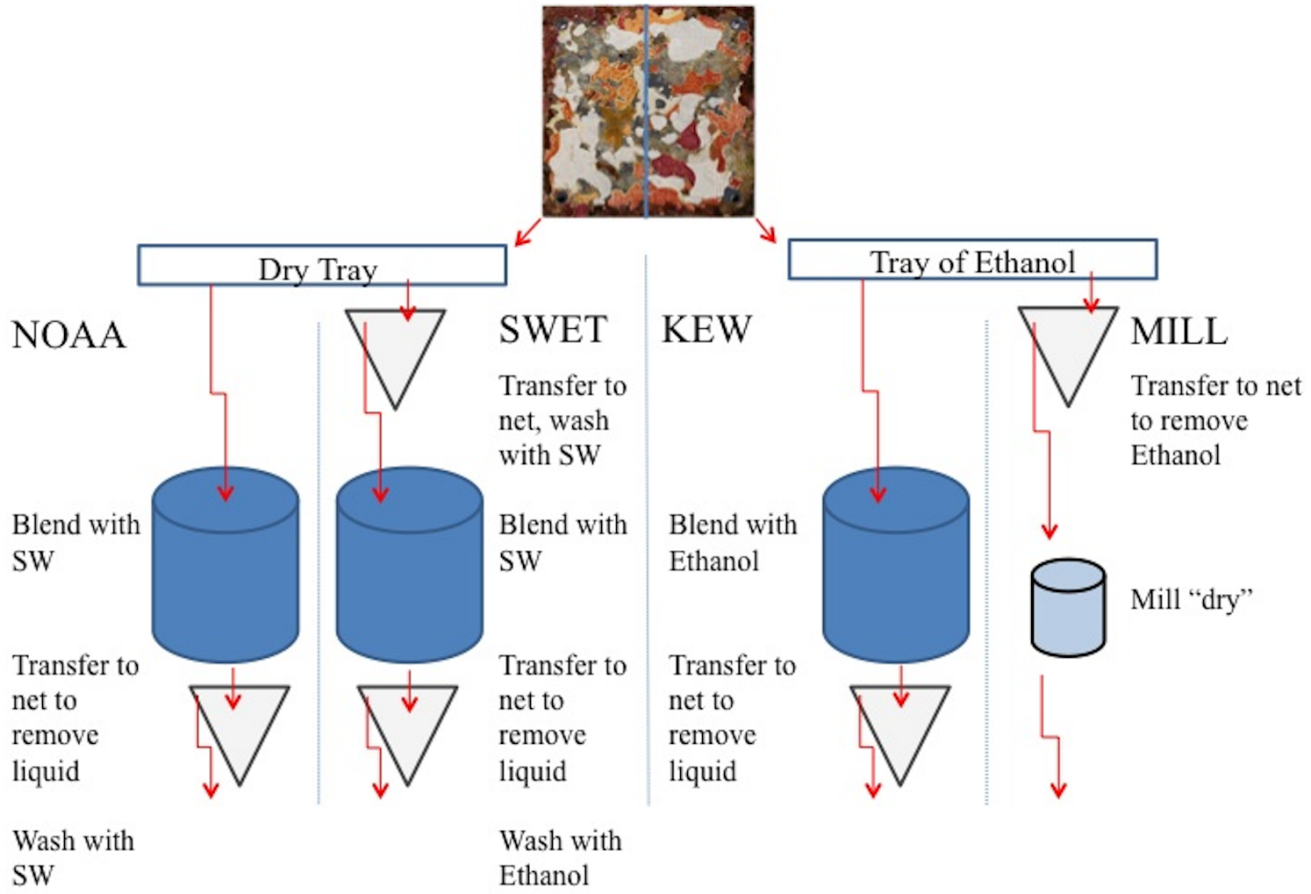
Diagram of four approaches employed to process sessile (attached) material from ARMS. All seawater (SW) was filtered through a sterilized 45 um nitex net, and each sample was blended for 1 minute at full speed using a household blender or ten 2-second strokes of an IKA A11 basic analytic mill (IKA Works, Inc., Wilmington, NC).

Unlike the motile fractions, four methods (KEW, NOAA, SWET, MILL) have been used or proposed to process the sessile fraction of the ARMS. All methods scrape the plates clean, homogenize the resulting material, and subsample for metabarcoding analysis. They differ broadly in the amount of ethanol and the filtering technique used (Fig 1). The KEW method relies on ethanol to immediately shut down nuclease activity after scraping, mimicking many standard preservation protocols. The NOAA method relies on FSW to wash and blend the sample and is currently employed on ships and in remote locations where chemical restrictions and transportation issues make the quantities of ethanol needed for other methods difficult. The SWET method repeats the NOAA method but includes a pre- and post- blend wash with FSW and EtOH, respectively, to attempt to remove impurities and terminate nuclease action. Finally, the MILL method aims to more finely and uniformly homogenize samples, especially heavily calcified organisms such as molluscs, and excludes post-blend filtering to avoid washing away DNA.

We generated four comparative scraped subsamples from each ARMS unit to examine method performance. The top and bottom of each ARMS plate (nine per unit) were photographed to document percent cover of dominant sessile phyla. Half of each plate was scraped into a dry tray, and the other half was scraped into a tray of ethanol (95%). Care was taken to split large organisms between the subsamples. The resulting material was mixed and split in half again to create the four subsamples representing each method. Downstream methods are shown in Fig 1; for detailed methods see S1 File).

Each resulting subsample (n = 4) was split in fourths again and preserved in 95% EtOH, 25% DMSO or RNAlater and stored at -20°C for 1 month before DNA extraction. The fourth subsample was immediately extracted without preservation.

### DNA metabarcoding

Total DNA was extracted from 10 g of the homogenized sessile tissue (n = 48) and 1 g of decanted (to remove sediment from organic fraction) crushed tissue from the 106–500 μm and 500 μm–2 mm motile fractions (n = 17; see Leray & Knowlton [18]). MO-BIO Powermax Soil DNA Isolation Kits were used according to the manufacturer’s protocol with the addition of 400 μg/ml Proteinase K and an overnight lysis step at 56°C and 200 rpm. All DNA extracts were further purified using MO-BIO PowerClean DNA Clean-Up Kits, quantified (Qubit dsDNA HS Kit), run on an agarose gel, and DNA quality investigated using ImageJ software.

A dual-indexing approach was used to multiplex all 65 samples across four Ion Torrent PGM sequencing runs using tagged COI PCR primers (mlCOIintF/ jgHCO2198; [26–27]) and eleven Ion Xpress barcode adapters (Life Technologies). PCRs were performed in triplicate on 10 ng of DNA, and metabarcoding libraries were prepared for the Ion Torrent PGM platform (Life Technologies). See S1 File for details. The metabarcode datasets were deposited in the Dryad Digital Repository (http://dx.doi.org/10.5061/dryad.d47fm).

### Sequence analysis

DNA sequences were pre-filtered by the Torrent Suite Software version 4.0.2 (Life Technologies) and cleaned and processed following Leray & Knowlton ([18]; see S1 File). BLASTn (task argument: blastn, word size = 11, minimum e-value = 1e^-20^) searches were performed on OTU representatives in GenBank, BOLD (Barcode of Life Data Systems) and a curated database of 16,679 CO1 sequences from the Mo’orea Biocode inventory (Biocode; [25]). To assess the accuracy of identifications based on BLAST searches, we tested the use of different cut-offs (sequence identity and query coverage) in providing accurate identifications from GenBank for sequences of 233 previously identified specimens across 16 animal phyla successfully amplified by Leray et al. [27]. The top nine hits for each query sequence were retained (for distribution of sequences across phyla see S1 Table). Based on these results, we classified BLAST matches to our OTUs as either high (≥ 97% identity and ≥ 85% coverage) or medium (≥ 85% identity and ≥ 85% coverage). A phylogenetic approach was implemented on all OTUs using the Statistical Assignment Package (SAP, [28]), which assigns OTUs to higher taxonomic levels in the absence of direct matches. We used ≥ 70% sequence identity and accepted taxonomic assignments above 90% posterior probability cutoff [18]. We recovered full taxonomic hierarchies for each BLAST hit, but focused on phylum level identifications to minimize misidentifications. OTUs that matched bacteria were removed. When a given OTU was identified by more than one method, we compared the phylum identifications and tabulated mismatches. Because different databases employ different taxonomic hierarchies, we identified mismatches due to conflicting taxonomies and altered the taxonomic hierarchies to a standard nomenclature, favoring BOLD nomenclature over NCBI (S2 Table).

### Image analysis of ARMS plates

High quality images of the ARMS plates were analyzed using Coral Point Count (CPCe; [29]) to determine the relative abundance of dominant macroscopic sessile groups: Anthozoa, Bivalvia, Bryozoa, Porifera, Rhodophyta and Tunicata. For both sides of each plate 225 points were analyzed and the percent cover of each sessile group was calculated for each ARMS. Results were compared to the percentage of sequence reads for each representative group to assess which processing and preservation method was most consistent with these visually discernable groups.

### Statistical analysis

Statistical analyses were performed using PRIMER-E6 (PRIMER-E Ltd., Plymouth, UK) [30–31] and MINITAB 6.0 (Minitab). In PRIMER-E, Bray-Curtis and Jaccard dissimilarity matrices were calculated for rarefied sequence data from sessile (n = 5899) and motile (n = 4996, 106–500 μm; n = 1229, 500 μm–2 mm) ARMS fractions. Data were visually explored using nonmetric multidimensional scaling (NMDS) and hierarchical clustering (group average), with SIMPROF tests to identify natural groupings of samples that were not defined *a priori* (1000 permutations, significance level 5%) and to examine similarities in phyletic composition between CPCe (image analysis) and sequencing data. To examine the importance of preservation and processing methods on community composition, PERMANOVA and ANOSIM analyses were conducted. Diversity was examined using the Shannon–Weaver (H’) metric; ANOVA tests were performed in MINITAB 6.0 (Minitab) to test for differences in diversity between treatments. All analyses were carried out on rarefied OTU abundance and richness data, with and without singletons. Singletons were removed prior to rarefaction. Data presented herein represent abundance data (square-root transformed), void of singletons; other analyses showed similar patterns unless otherwise stated. To further investigate sessile differences, OTUs were merged by phylum and analyses described above were repeated. A SIMPER analysis was used to identify phyla that contributed most prominently to group differences (90% cut-off). To investigate differences in richness of sessile phyla across treatments, rarefied data were converted to presence and absence values, OTUs were merged by phylum, standardized by total, and square-root transformed before downstream analysis.

## Results

### Sequence data and bioinformatics

Using 233 previously identified barcoded specimens [27], we conducted an *a priori* test to assess an 85% cutoff (identity and query coverage) and to establish how well Genbank is populated for identifying marine taxa at the phylum, class, and order level. Only 65 (28.8%) matched a Genbank entry at ≥ 97% identity / ≥ 85% coverage and were highly taxon dependent. All but one of the Chordata matched a Genbank entry (13 of 14), whereas none of the representatives of Bryozoa, Platyhelminthes, or Nemertea matched ≥ 97%. A relaxed threshold of 85% identity (≥ 85% coverage) increased our match rate to 62.2% (an additional 33.4%, 145 total matches) and only one query matched to an incorrect phylum in Genbank. Analysis of class and order level matches at ≥ 85% revealed 3.5% and 8.3% incorrect identifications, respectively (S1 Table). As a result, we used the relaxed threshold for phyla identification and analyses.

A total of 3,372 OTUs were recovered from 1,228,070 sequences across all subsamples and size fractions (106–500 μm and 500 μm–2 mm motile fractions and sessile fraction). After removing 916 singletons, 484 of the remaining OTUs matched reference sequences at ≥ 97% similarity and ≥ 85% query coverage in taxonomic databases (GenBank, BOLD or Biocode; see Table 1). These matches account for 38.6% of the total sequences. The local Biocode database accounted for 302 (62.4%) of these OTU matches and 86.9% of the sequence matches. An additional 304 (12.4%) of the total OTUs and 47.7% of the total sequences could be identified to a higher taxonomic level based on sequence identities between 85–97% (≥ 85% query coverage), thereby increasing the number of OTUs identified to 788 (32.1%), which account for 86.3% of the total sequences (Table 1).

**Table 1:** Summary of amplicon data across sessile and motile (106–500 μm and 500 μm–2 mm) ARMS fractions (* ≥ 85% sequence similarity in BOLD, NCBI or Biocode). All identifications are made ≥ 85% query coverage. Data excludes singletons.

	106–500μm	500μm–2mm	Sessile	Total
Sequences	85,384	117,119	1,024,651	1,227,154
Total no. of OTUs	1,033	627	2,228	2,456
OTUs with ID (> 97%)	29.1%	39.1%	18.4%	19.7%
Sequences with ID (> 97%)	39.0%	62.3%	35.9%	38.6%
OTUs with ID (> 97%) from Biocode only	19.0%	23.2%	11.5%	12.3%
Sequences with ID (> 97%) from Biocode only	31.8%	50.7%	31.7%	33.5%
OTUs with any match*	44.9%	59.5%	30.5%	32.1%
Sequences with any match*	57.8%	90.1%	88.2%	86.3%
OTUs match with SAP only	25.7%	18.5%	22.7%	22.8%
Sequences match with SAP only	15.5%	3.2%	3.3%	4.2%
OTUs unknown	29.4%	22.0%	46.8%	45.1%
Sequences unknown	26.8%	6.6%	8.5%	9.6%
Rarefied sequences/ sample	4,996	1,229	5,899	
Rarefied OTUs	856	350	1,751	

Because we used multiple databases, we investigated phylum level OTU mismatches. All but 15 of 419 (3.6%) cases and two (1.2%) of 166 cases with more than one match at the medium and high thresholds, respectively, produced the same identification at the phylum level. In downstream analyses, mismatched OTUs were classified as “ambiguous”.

The Statistical Assignment Package (SAP) assigned identities to 1125 OTUs (45.8%), half of which were identified only by SAP. We tabulated mismatches at the phylum level between the 564 OTUs identified both by SAP and via our BLAST criteria (85%/85%) to assess the ability of SAP to correctly bin sequences into the correct phylogenetic space using publically available sequences from Genbank. Of these OTUs, 78 (13.8%) had conflicting identifications at the phylum level. Close examination of these mismatches revealed that 53 (9.7%) were confidently identified by the Biocode database (sequences currently not available to SAP analyses) and therefore, likely erroneous identifications via SAP, with 25 having been wrongly assigned to Insecta. Sequences that could not be identified via BLAST or by using SAP accounted for 45.1% of the OTUs but only 9.6% of the sequences.

### Mo’orea reef diversity and community overlap across ARMS fractions

**Fig 2:**
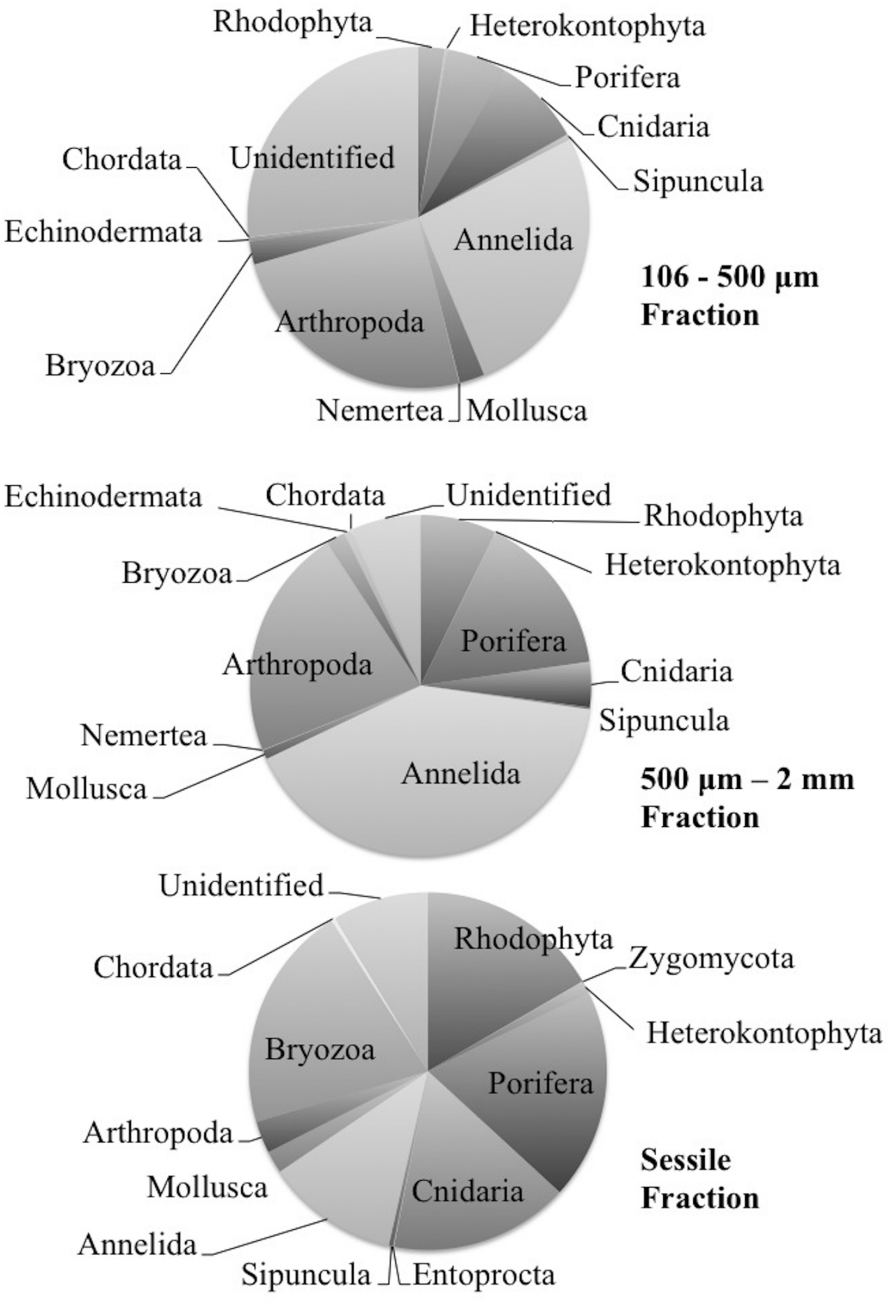
Pie charts showing relative abundance of phyla in each ARMS fraction. Dominant phyla are labeled; for details of less abundant phyla please see supplementary material.

Based on identified OTUs, ARMS contained 28 phyla, including 17 animal, three algal, five protozoan, and three fungal phyla. The top ten OTUs made up 64.7% of the sequences and the 50 most abundant OTUs in each fraction were readily distinguishable and biologically sensible. The large motile fraction (500 μm– 2 mm) was dominated by arthropods (n = 17) and annelids (n = 17) and only 3 OTUs were not identifiable (Fig 2). Similarly, the small motile fraction (106–500 μm) was characterized by a large number of arthropods (n = 14) and annelids (n = 14), but contained 13 unidentifiable OTUs. Both of these motile size fractions yielded sessile phyla such as Rhodophyta, Bryozoa, and Porifera, probably because small portions of sessile taxa broke off during disassembly and were trapped by the sieves. The sessile fraction was dominated by sponges (n = 7), rhodophytes (n = 7), bryozoans (n = 6), and cnidarians (n = 5), but also yielded taxa such as annelids (n = 5) and arthropods (n = 3), which can be either sessile or motile, and 11 unidentifiable OTUs (Fig 2; S3 Table).

### Motile preservation experiment

Motile fractions (106–500 μm and 500 μm–2 mm fractions) from the three recovered ARMS units were subsampled into three preservatives (EtOH, DMSO and RNAlater) and sequenced to investigate compositional shifts in the bulk motile community. Of the 18 total samples (3 ARMS x 2 motile fractions x 3 preservation techniques), one 106–500 μm sample was lost in transport. All samples returned over 100 ng/μl DNA after DNA extraction with > 40% percent of DNA > 1000bp (S4 Table). Examination of retrieved community composition showed no significant differences between preservation methods in OTU and phylum analyses for both fractions (PERMANOVA/ANOSIM analyses; S2 File). Community composition was significantly different across the three ARMS, but no significant differences were found in any pairwise tests for either fraction (ANOSIM).

### Sessile processing and preservation experiment

**Fig 3:**
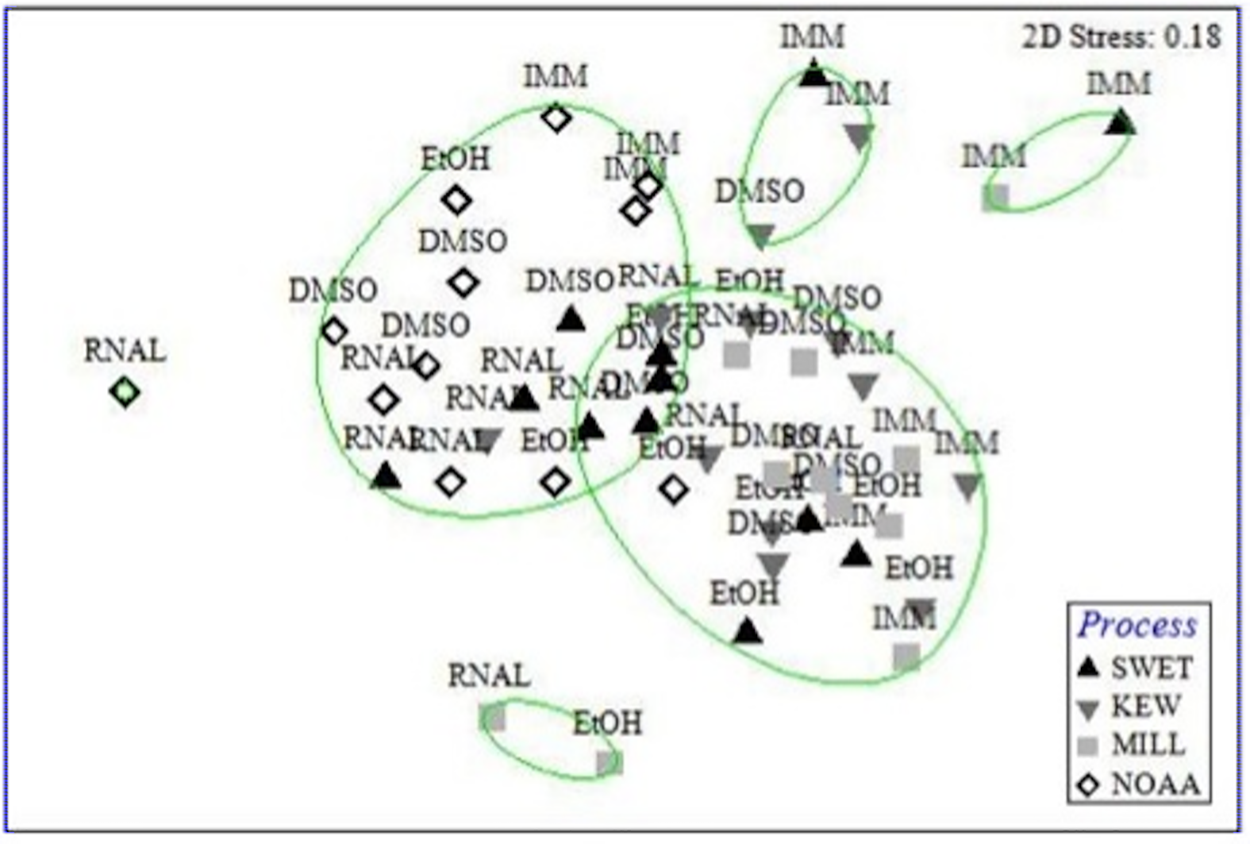
Multidimensional scaling of the sessile eukaryotic community (OTU abundance data; no singletons) retrieved from three ARMS using four processing methods (NOAA, SWET, KEW and MILL) and four storage techniques: 1 month at -20°C in EtOH, DMSO or RNAlater, or samples were immediately extracted (IMM). Clusters represent similarity between samples (50%), based on a Bray-Curtis similarity matrix.

Forty-eight samples (3 ARMS x 4 processing methods x 4 preservation techniques) were sequenced to investigate biases in the resulting composition of the bulk sessile community (Fig 3). Ethanol (EtOH) preserved samples frequently returned both low quantities of DNA (< 100 ng/ul DNA; 66.7%) and < 20% high quality DNA (>1000 bp; 58.3%). In contrast, DMSO preserved samples returned high DNA quantity and quality (S4 Table). RNAlater stored samples also returned good quality DNA, but not always in high quantities. Between processing methods, MILL samples frequently returned low quantities of DNA (50%) and KEW and SWET samples most frequently returned low quality DNA (25%). NOAA samples returned the highest quality of DNA (S4 Table).

#### OTU community composition and diversity

Significant differences in OTU community composition were found between ARMS, processing method, and preservation technique (see S5 and S6 Tables for PERMANOVA and ANOSIM statistics). In pairwise comparisons, significant differences were seen between NOAA processed samples compared to all other methods and between SWET and MILL methods (S6 Table). When partitioned by preservation, significant differences were only found within DMSO preserved samples (Tables A and C in S3 File). An analysis of Shannon diversity (H’; by preservation subset) showed significant differences within DMSO stored samples (ANOVA F = 6.86, p < 0.013), with NOAA processed samples being significantly more diverse than MILL samples (Tukey tests; see S7 Table for diversity metrics).

For preservation technique, significant community differences were observed in pairwise comparisons (ANOSIM) between immediately extracted samples and all preservation techniques and between RNAlater and EtOH preserved samples (S6 Table). When partitioned by processing method, ANOSIMs revealed that these differences were significant within NOAA and SWET processed samples; (Table B in S3 File). No significant differences between preservation techniques were found with richness data (Table C in S3 File) or Shannon diversity (H’; ANOVA).

#### Phyla community composition and diversity

**Fig 4:**
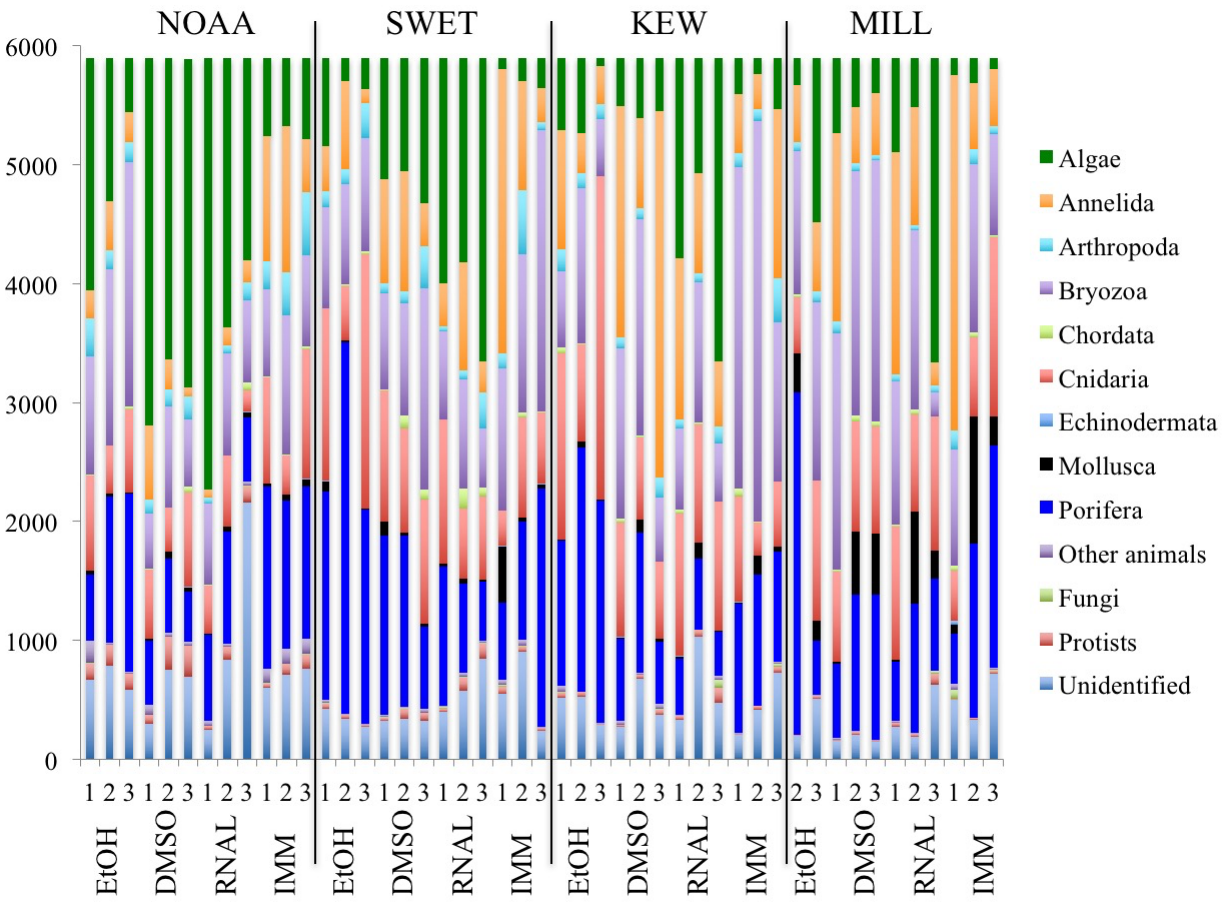
Proportion of sequences belonging to each phylum (abundance data) retrieved from three ARMS using four processing methods (SWET, KEW, MILL, and NOAA) and four storage techniques: 1 month at -20°C in EtOH, DMSO, or RNAlater (RNAL), or samples were immediately extracted (IMM). The category “Other animals” represents Hemichordata, Entoprocta, Rotifera, Tardigrada, Xenacoelomorpha, Gastrotricha, Nemertea, Platyhelminths, Sipuncula and Nematoda.

Significant differences in phyla composition and richness were found between ARMS, processing method, and preservation technique (S8 and S9 Tables). Pairwise comparisons of processing methods matched OTU analyses, but significant differences between processing methods were also found in EtOH stored samples (S9 Table and Table A in S4 File). Pairwise comparisons of preservation methods showed significant differences between immediately extracted samples and DMSO/RNAlater preserved samples and between EtOH and RNAlater/DMSO preserved samples (S9 Table). Data partitioning by processing method found significant differences in preservation technique within all processing methods (except MILL; Table B in S4 File). The relative abundance of phyla across ARMS and processing and preservation methods is shown in Fig 4.

In a SIMPER analysis, NOAA and KEW/MILL methods had the greatest compositional shifts, which translated into differences in particular phyla (Table A in S5 File). Rhodophytes (NOAA samples) were the main driver of these differences (contributing 20.3 and 18.4%, respectively), but annelids (KEW/MILL samples; 16.3 and 11.2%, respectively) and various other phyla also contributed. An analysis partitioned by preservation technique confirmed these differences (Table B in S5 File). Richness data showed similar patterns, suggesting that the presence of phyla and not just their abundance were driving the differences observed; unknown OTUs in NOAA samples were important in driving differences here (8.5 and 8.3%, respectively).

A SIMPER analysis comparing preservation techniques revealed greatest dissimilarity between RNAlater and EtOH/immediately extracted samples, with the Rhodophytes as an important driver (RNAlater samples; contributing 19.8 and 22.9%, respectively), however various other phyla also contributed (Table A in S5 File). Partitioning data by processing method confirmed these differences (Table B in S5 File), as did richness data. However, richness data revealed that fewer Arthropoda and Annelida sequences and greater unknown OTUs and Heterokontophyta (RNAlater samples) were driving observed differences.

Due to the importance of Rhodophyta in methodological differences, analyses were repeated after removing the phylum from the dataset (with re-rarefied data). The removal of rhodophytes did not eliminate dissimilarities between processing methods, suggesting Rhodophyta were not the sole driver of differentiation (PERMANOVA/ANOSIM; Tables A and B in S6 File). However, phylum-level differences in preservation technique were weak (ANOSIM Global R = 0.073, P<0.05) and ANOSIMs partitioned by processing method revealed no differences between preservation techniques (Tables A and B in S6 File). SIMPER analysis showed unidentified OTUs (NOAA samples) and annelids (MILL/KEW samples) to be driving differences between NOAA/MILL (13.1 and 12.3%, respectively) and NOAA/KEW (11.0 and 15.5%, respectively) samples.

**Fig 5:**
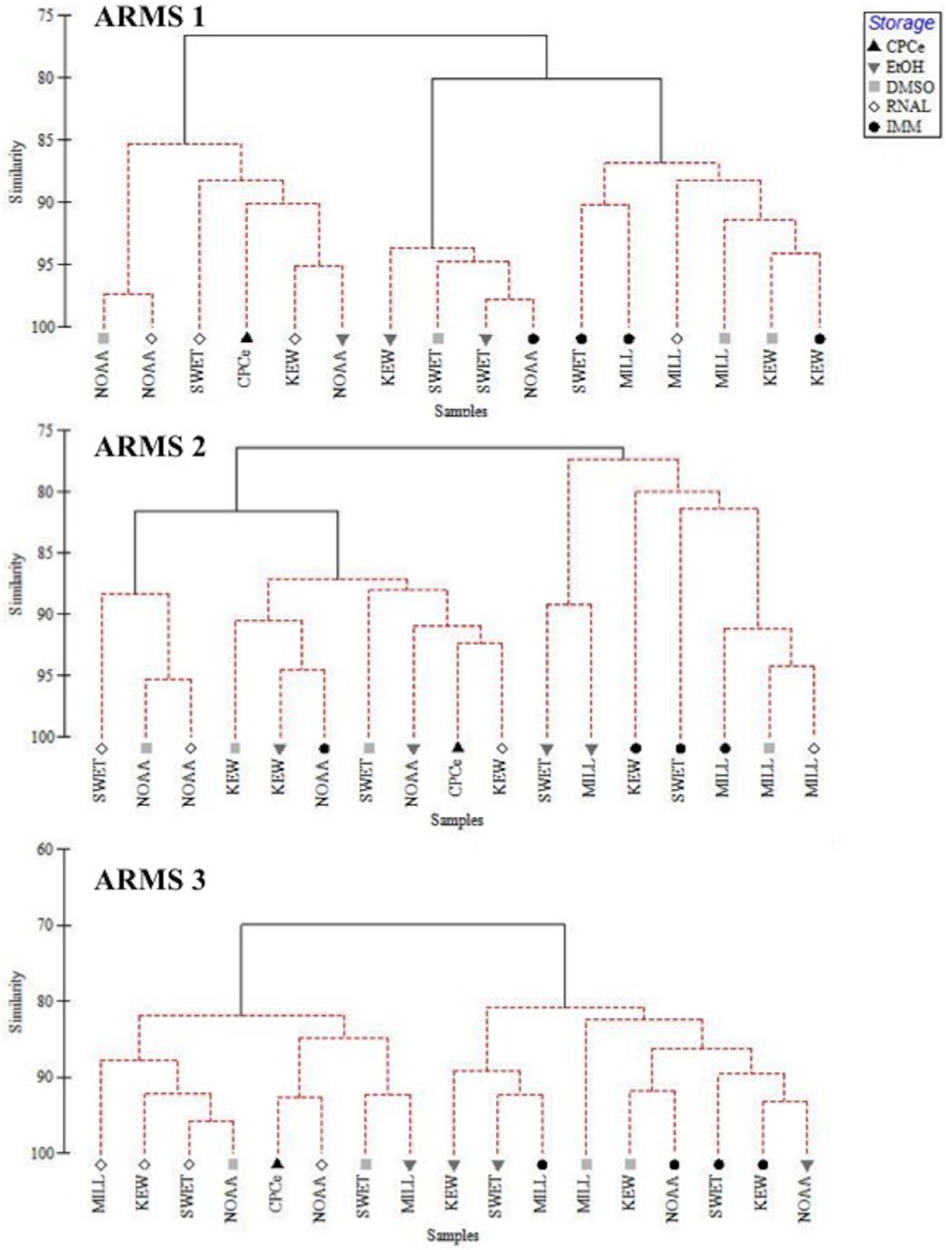
Group average hierarchical clustering with SIMPROF tests (red bars) of the community found on three ARMS via NGS and CPCe. Community data are based on the percentage of the sessile community belonging to Rhodophyta, Chlorophyta, Porifera, Tunicata, Bryozoa, sessile Mollusca and Anthrozoa. Samples represent different processing methods (NOAA, SWET, MILL and KEW), different storage methods (EtOH, RNAlater, DMSO and Immediate extraction of DNA (IMM)) and image analysis of each overall ARMS (CPCe). Clustering is based on a Bray-Curtis similarity matrix of relative percentages of each group.

Cluster analyses with SIMPROF tests comparing the CPCe image analysis of phyla with the relative abundance of phyla in sequencing data showed a number of groupings within each ARMS unit (Fig 5). Data showed NOAA samples and RNAlater preserved samples clustering more often with CPCe data followed by those preserved with DMSO (Fig 5). SIMPER analysis revealed that clusters containing CPCe data had higher percentages of Rhodophytes compared to far right clusters (Fig 5), which were characterized by increases in Bryozoa (ARMS1&3), Porifera (ARMS2&3) and Bivalvia (ARMS2; S10 Table).

## Discussion

This investigation of cryptic diversity in a small area of Mo’orea coral reefs yielded impressive diversity using metabarcoding techniques, with the recovery of nearly 1300 OTUs (n = 1293) per square meter and 28 phyla, including at least 17 of 33 marine metazoan phyla over 2.607 m^2^ of examined “reef”. The Mo’orea Biocode Project recovered only 3591 species from sampling all the island’s marine habitats over five years, but the focus was only on species >2mm. These results demonstrate the magnitude of diversity in species of small size. The advancement of metabarcoding techniques combined with the decline in high-throughput sequencing costs and the use of standardized sampling methods, such as ARMS, provides a mechanism in which to characterize benthic communities across all species, regardless of size. However, global comparisons are only valid if the data are gathered in comparable ways. This study verified the need for standardization of ARMS protocols, documented the handicaps of current bioinformatics methods in retrieving accurate taxonomic identification, and demonstrated the importance of having more comprehensive curated COI databases and inventories.

### Importance of methodological standardization

The availability of metabarcoding techniques and divergent methodologies across studies have instigated the standardization of processing and preservation protocols for the assessment of naturally occurring communities [32]. However, the vast majority of these protocols involve bacterial community assessments e.g. [33–35] and have yet to tackle assessments of complex eukaryotic communities.

#### Processing of the ARMS sessile fraction

Overall, these results demonstrate that minor variations in field protocols result in different community profiles, making it imperative to standardize methods used within and across studies to ensure that measurements of biodiversity are temporally and spatially comparable. We speculate that MILL differences are partly attributed to its ability to more effectively break down the calcium carbonate shells and exoskeletons of molluscs, bryozoans, and annelids. NOAA samples had significantly higher Shannon diversity (H’) than KEW/MILL samples. The addition of filtered (45 μm) water in SWET/NOAA methods may add environmental DNA (eDNA; [36]) to the subsample, which could increase diversity and the importance of unknown OTUs, as seen in NOAA samples. However, no difference in community composition or diversity was seen between KEW (which does not use water) and SWET (which does) methods, making this less likely as the sole reason for these differences. Furthermore, in a SIMPER analysis (richness data), the contribution of each phylum to the differences was low, suggesting that a range of phyla contribute to compositional shifts.

We recommend the NOAA method for processing the sessile fraction in future ARMS studies. While differences between methods are apparent, it is more difficult to determine which method gives the most accurate representation of the community. Our analysis of the main sessile groups on the ARMS (Rhodophyta, Porifera, Bryozoa, Tunicata and Bivalvia), determined by image analysis and sequencing data shows that NOAA samples more frequently clustered with or adjacent to image analysis (CPCe) profiles. In contrast, MILL samples rarely clustered with CPCe data. While this analysis is not conclusive for one method, these overall patterns suggest that the NOAA method is more accurate in assessing macroorganism abundance. In addition, DNA extractions of NOAA processed samples return the highest quality of DNA compared to other methods and low DNA quantities were only retrieved from EtOH and RNAlater preserved samples. As the NOAA method is simple, cheap, and requires a limited quantity of chemicals, it would also be the easiest to implement globally, on ships, in remote field locations and on projects with limited budgets. The absence of EtOH would additionally allow for microbial and viral sampling of this material, with the addition of 0.22 μm or TFF filtered water [37], respectively.

#### Preservation of ARMS fractions

Regardless of processing method, we show that preservation method has an impact on the retrieved sessile community profile. Results show largest differences between immediately extracted samples and those stored for one month, suggesting that storage in any buffer affects the community retrieved. As immediate extraction is impossible at many ARMS sites, we do not suggest this as standard protocol.

The superiority of DMSO over EtOH has been shown previously [18, 38]; after one month of storage, we confirm these patterns with more degraded and lower DNA quantity from EtOH preserved samples. In contrast, DMSO preserved samples returned no low quantity extracts and had better DNA quality than other preservatives. RNAlater preserved samples consistently clustered with CPCe data for the limited phyla tested, clusters that were driven by a higher abundance of Rhodophyta. While fewer DMSO preserved samples clustered with CPCe results, ANOSIM analysis showed no significant differences between RNAlater and DMSO preserved samples when assessing the entire community or when comparing diversity. Gray et al. [33] also showed similar microbial profiles when using these buffers, determining that neither preservative outperformed the other. However, here we see some evidence for the retrieval of low quantities of DNA in RNAlater preserved samples. Furthermore, NOAA processed, DMSO preserved samples clustered with or adjacent to CPCe data in all three ARMS tested. As such we suggest DMSO buffer for preserving sessile ARMS material, with the caveat that performance of storage buffers over longer periods of storage (>1 month) is not known. Balancing the need for accurate data with finances and chemical restrictions, DMSO buffer is also preferred as it is less expensive (compared to RNAlater), is not a restricted chemical (compared to ethanol) [39] and provides high molecular weight DNA [18], which will be important as read lengths of sequencing platforms increase.

We suggest that while sequencing of the sessile fraction is imperative for assessments of biodiversity in regional studies and that using one method of preservation of the motile fraction is preferable, the motile fractions may provide more reliable and replicable estimates of biodiversity for global comparisons. For the motile fauna, this study shows there is no significant difference among the three commonly used preservation methods for the 106–500 μm and the 500 μm– 2 mm motile fractions and that DNA quality and quantity are stable across preservatives. These results contrast with the preservation of the sessile fraction, most likely due to the lack of disruption of metazoan tissue prior to storage.

### Importance of taxonomic identifications to improve meta-barcoding analysis on coral reefs

This study demonstrates the need for more comprehensive inventories of cryptic species and curated databases. In our test data set, which includes a range of marine phyla from coral reefs (previously published in Leray et al. [27]), only 28.8% of OTUs retrieved high threshold matches (≥ 97% identity and ≥ 85% coverage) in publically available databases (Genbank and BOLD). These databases are sparsely populated with important coral reef taxa, such as Bryozoa and Annelida, making a significant proportion of ≥ 97% identifications impossible. High threshold matches in our data only account for 7.4% of OTUs and 5.1% of sequences using these databases. Similar results have been found from other marine ecosystems, such as oyster reefs, where < 12% of sequences from ARMS could be identified at ≥ 97% identity [18]. As a result, the vast majority of sequences from metabarcoding studies will remain unidentified if databases are not improved.

Our limited ability to identify coral reef taxa can be overcome by scalable, standardized and quasi-automated (e.g. molecular) approaches as seen in this study, along with local and regional inventories of cryptic reef species. Several large-scale DNA-barcode initiatives have targeted coral reefs since 2006, in the Pacific (Biocode) and in the Indian Ocean (BIOTAS Project). While expensive and time consuming, these efforts are essential for characterizing reef diversity.

This study highlights the urgent need for targeted investigations of benthic meiofauna (small metazoans between 45–500 μm) in future inventories. These small size fractions are often diverse [40], and represent a major portion of the unidentifiable OTUs, and of marine biodiversity [41]. Meiofaunal assemblages perform essential roles in marine ecosystem processes, namely nutrient cycling, secondary production, sediment transport and mineralization [42], and need to be better characterized [43].

### Current limitations of databases

While the coral reef community waits for identifications based on large- and small-scale inventories to make their way into publically available databases, it is important to understand the limitations of current identification methods. Using a Bayesian phylogenetic approach, the Statistical Assignment Package [28] is an important tool, providing a large number of low taxonomic resolution identifications for sequences that would otherwise remain unidentified using BLAST. In this study, SAP-only identifications account for 22.8% of the 2,456 OTUs. Testing a further 23% of our data that had BLAST and SAP identities revealed that 13.8% of OTUs had conflicting identifications at the phylum level. Close examination revealed that 67.9% of these were confidently identified by the Biocode database (sequences currently not available to SAP analyses) and, therefore, likely erroneous identifications via SAP. Thus, even though the phylogenetic approach employed in SAP could be considered conceptually superior to BLAST, often it appears to erroneously assign COI sequences to phylum with high posterior probability in the absence of relatively close matches in a reference database. Indeed, more than 40% of the SAP identifications deemed erroneous were classified as insects. While sequences only identified by SAP equal 4% of our dataset, without the use of a local database, like Biocode, to identify many of our sequences, this number would be much larger.

We tested the use of an 85% identity threshold (and coverage ≥ 85%) to give accurate identifications at higher taxonomic ranks (phyla, class, and order) and found that 144 out of 145 known queries were correctly identified to phylum, and over 95% of these were correctly identified at the class level. Thus, in the absence of sufficiently populated databases, marine community analyses could use this cut-off to understanding of the distribution of COI OTUs across phyla in comparative diversity studies. This method is computationally faster than using phylogenetic approaches such as SAP. Using these medium threshold matches is likely to remain important until many more species have high quality barcodes in publically available databases.

## Conclusions

ARMS provide an opportunity to compare benthic diversity on a global scale, investigate cryptic species not quantified in traditional biodiversity surveys, contribute to the ongoing debate regarding centers of biodiversity, and increase our understanding of ecosystem responses to anthropogenic impacts. However, to use these invaluable data to their full potential, the processing of ARMS needs to follow a standardized method globally that is cost effective, feasible in remote field locations, and provides an accurate picture of the community. Scientists also need to address the limitations that result from sparsely populated databases, renew interest in targeted exploration of understudied taxa, and ensure that high quality barcodes for identified species make it into public databases quickly.

While we focus on ARMS, the effect of small variations in field protocols on the outcome of eukaryotic diversity assessments has yet to be sufficiently tackled for other large efforts to sample biodiversity on a global scale. With the growth of metabarcoding and other environmental DNA approaches to assessing and monitoring biodiversity, emphasis on standardization of protocols is critical to ensure comparability. Various handling, processing, preservation, and extraction procedures of all mixed libraries (e.g. fish guts, plankton tows, malaise traps, meiofauna) likely create biases. ARMS are not an exception and likely the rule. If we are to leverage global efforts to genomically characterize global biodiversity [44–46] all sampling programs should publish detailed, standardized procedures, e.g. [47] to ensure that the data generated persist far beyond the particular study for which it was initially used. We hope that these findings will catalyze the careful replication of other field techniques for future diversity assessments.

## Supporting information

S1 File Detailed methodologies. (PDF)

S2 File PERMANOVA and ANOSIMs for motile preservation experiment OTU data (Table A and C, respectively), and data merged by phylum (Table B and D, respectively). (PDF)

S3 File One-Way ANOSIMs and Tukey Tests showing differences in community composition retrieved by processing method (OTU data; Table A), preservation method (OTU data; Table B) and differences in community richness retrieved by preservation and preservation method (OTU data; Table C). (PDF)

S4 File One-Way ANOSIMs and Tukey tests showing differences in community composition retrieved by processing method (Table A) and preservation method (Table B). (PDF)

S5 File SIMPER analysis showing phylum level differences in community composition across all data from the sessile processing experiment, as retrieved by processing method (Table A; top table) and preservation technique (Table A; bottom table) and partitioned by preservation method (Table B; top two tables) and processing method (Table B; bottom two tables) from the sessile processing experiment, to provide more detail of the important phyla. (PDF)

S6 File One-Way ANOSIMs and Tukey tests showing differences in community composition retrieved by processing and preservation method, void of Rhodophyta, for OTU data (Table A) and data merged by phylum (Table B). (PDF)

S1 Table Accuracy of relaxed parameters (≥85% sequence similarity) when assigning to higher-level taxonomic ranks with Blastn. Data shown represent phyla distribution among queries.
(PDF)

S2 Table Conflicting and altered taxonomies in database searches. (PDF)

S3 Table Phylum level identifications of OTUs and their relative abundance by ARMS fraction. Bold text represents most abundant phyla in each fraction.
(PDF)

S4 Table Raw data (top tables) and overall summary (bottom table) of the quantity of DNA and the percentage of that DNA that is > 1000 bp (as determined by ImageJ analysis) recovered from DNA extractions of sessile and motile ARMS samples across different processing and preservation methods. Bold values indicate low DNA quantity (<100 ng/ul) or quality (<20% >1000bp). Gel images were not available for immediately extracted samples. In the bottom table, values in bold indicate the processing and preservation methods with highest (bottom two rows) and lowest (top two rows) overall DNA quantity and quality.
(PDF)

S5 Table PERMANOVA for sessile processing experiment (OTU data). (PDF)

S6 Table ANOSIMs and Tukey Tests for sessile processing experiment (OTU data). ANOSIMs were carried out across all data for ARMS, processing method and preservation method. Tukey tests reported were calculated from all abundance data (* p ≤ 0.005, ** p ≤ 0.05, *** p > 0.05).
(PDF)

S7 Table Diversity data for sessile processing experiment, showing number of OTUs (S), species richness (Margalef; d), Pielou's eveness (J'), and Shannon diversity (H'). Based on rarefied abundance data, void of singletons.
(PDF)

S8 Table PERMANOVA for sessile processing experiment, data merged by phylum. (PDF)

S9 Table ANOSIMs and Tukey Tests for sessile processing experiment, data merged by phylum. ANOSIMs were carried out across all data for ARMS, processing method and preservation method. Tukey tests reported were calculated from all abundance data (* p ≤ 0.005, ** p ≤ 0.05, *** p > 0.05).
(PDF)

S10 Table SIMPER analysis of the subset of the communities retrieved from the sessile processing experiment, used in comparison with CPCe (Coral Point Count) data. Tables show phylum level differences in community composition between SIMPROF clusters, which compared community composition data retrieved from different processing methods and CPCe image analysis, for all three ARMS (Fig 5). * represents the cluster containing the community as determined by CPCe image analysis. Values to the left of the tables show within group similarities for comparison with between group average differences. Percentage contributions of the most important phyla to community differences are reported with brackets to indicate which cluster returned the higher value for each phyla.
(PDF)
