# Supplementary material for: The importance of standardization for biodiversity comparisons: A case study using autonomous reef monitoring structures (ARMS) and metabarcoding to measure cryptic diversity on Mo’orea coral reefs, French Polynesia
Source: PLoS One. 2017 Apr 21;12(4):e0175066. doi: 10.1371/journal.pone.0175066 (PMC5400227; doi:10.1371/journal.pone.0175066)
Supplement: S1 File — (PDF) [file pone.0175066.s001.pdf]

S1 File. Detailed methodologies.

Detailed methodologies for processing of ARMS sessile fraction for metabarcoding analysis:

ARMS were disassembled and sessile material was scraped from each plate, as discussed in the methods section. NOAA and SWET methods call for material to be scraped into a dry try, while KEW and MILL methods call for material to be scraped straight into 95% ethanol. Once material was scraped into these trays and divided in two, SWET and MILL subsamples had a pre-blend rinse (Table 1). For SWET, material was rinsed with filtered seawater (FSW), squeezed dry and placed in the blender. For MILL, material was placed in the net to remove excess ethanol. All SW was filtered through a sterilized 45 μm nitex net.

Next each subsample was blended for 1 minute at full speed using a household blender (NOAA, SWET, KEW) or ten 2-second strokes of an IKA A11 basic analytic mill (MILL; IKA Works, Inc., Wilmington, NC). For NOAA and SWET, FSW was added before blending (40 ml). For KEW, material was blended with original ethanol (~ 40 ml); MILL samples were milled dry. Blended material was placed within a 45 μm nitex net and rinsed according to indicated post-rinse blend (Table 1). For KEW, the homogenate was squeezed within the net to remove access ethanol and then subsampled. For the NOAA method, homogenate was rinsed with FSW until clear at which point material was subsampled. For SWET, additional ethanol was applied to the homogenate within the net and filtered through before subsampling. The homogenate within the mill was directly subsampled. See Table A for overview of methods.

Table A. ARMS sessile processing methods currently employed and the associated differences among them (SW = filtered (45 µm) sea water; EtOH represent 95%).

**Method**	**Scraping Medium**	**Pre-Blend Rinse/Filter**	**Blend**	**Post-Blend Rinse**
NOAA	Dry	N/A	SW	SW
SWET	Dry	SW	SW	EtOH
KEW	EtOH	N/A	EtOH	N/A
MILL	EtOH	Squeeze dry	N/A	N/A

Detailed methodologies for PCR and Ion Torrent library preparation:

PCRs were performed on 10 ng of DNA, as determined by dsDNA HS assays (Invitogen) on a Qubit fluorometer. DNA was combined with 12.2 µl dH2O, 1 µl of each primer (10 µM stock), 1.4 µl of 10 mM dNTPs, 0.4 µl of Advantage 2 Polymerase (Clontech) and 2 µl Advantage 2 DNA buffer (Clontech). PCR conditions were as follows: 1 cycle at 95 °C for 10 min; 16 cycles at 95 °C for 10 s, 62 °C for 30 s (decreasing by 1 °C every cycle) and 72 °C for 1 min; 20 cycles at 95 °C for 10 s, 46 °C for 30 s and 72 °C for 1 min and one final extension at 72 °C for 7 min. Each PCR was conducted in triplicate.

Following amplification, PCR triplicates were pooled, cleaned using Agencourt AMPure XP beads, diluted 1:5 and quantified. Equimolar amounts of DNA from 7 PCR pools (with 7 different primer tags) were combined. The Ion Plus Fragment Library Kit (Life Technologies) was used to end-repair DNA fragments and ligate Ion Xpress barcode adaptors, using the manufacturer's instructions. qPCR (Kappa Biosciences) accurately quantified DNA for final

pooling of libraries, and emulsion PCR was carried out on the Ion One Touch 2 using the OT2 400bp kit (Life Technologies). Enriched ISPs were sequenced on the Ion Torrent PGM platform using the 400 bp v2 kit (Life Technologies), allowing for sequencing of the full target fragment.

Detailed methodologies for sequence processing:

DNA sequences were pre-filtered by the Torrent Suite Software version 4.0.2 (Life Technologies) and demultiplexed in Mothur [1] based on primer tail and Ion Xpress barcode. Sequences that were *(i)* shorter than 250 bp, *(ii)* had more than two mis-matches in either primer sequence, *(iii)* had any homopolymer region longer than 8 bp or *(iv)* had any ambiguous base calls, were removed from the dataset. Chimeras were checked and removed in Mothur. Multiple Alignment of Coding Sequences (MACSE; [2]) was used to align reads to a high-quality library of CO1 barcodes from the Mo'orea Biocode Project (http://mooreabiocode.org/) to remove sequences that had *(i)* stop codons (using invertebrate mitochondrial translation table-5 and flatworm translation table-14), *(ii)* frame shifts, *(iii)* insertions and *(iv)* more than 3 deletions (Leray et al. 2012). This high-quality library contains 7,675 sequences from 30 animal phyla from an all-taxa biodiversity inventory of the Moorea Island Ecosystem [3]. Clustering of unique sequences was performed using a Bayesian approach with Clustering 16S rRNA for OTU Prediction (CROP; [4]).

References

1. Schloss PD, Westcott SL. Introducing mother: A computation toolbox for describing and comparing microbial communities. Available: http://www.mothur.org/ ; 2009.

2. Ranwez V, Harispe S, Delsuc F, Douzery EJP. MACSE: Multiple Alignment of Coding SEquences accounting for frameshifts and stop codons. PLoS ONE. 2011;6(9):e22594.
3. Leray M, Yang JY, Meyer CP, Mills SC, Agudelo N, Ranwez V et al. A new versatile primer set targeting a short fragment of the mitochondrial COI region for metabarcoding metazoan diversity: Application for characterizing coral reef fish gut contents. Front Zool. 2013;10(34):1-14.
4. Hao X, Jiang R, Chen T. Clustering 16S rRNA for OTU prediction: a method of unsupervised Bayesian clustering. Bioinformatics. 2011;27(5):611-618.
