# Supplementary material for: The importance of standardization for biodiversity comparisons: A case study using autonomous reef monitoring structures (ARMS) and metabarcoding to measure cryptic diversity on Mo’orea coral reefs, French Polynesia
Source: PLoS One. 2017 Apr 21;12(4):e0175066. doi: 10.1371/journal.pone.0175066 (PMC5400227; doi:10.1371/journal.pone.0175066)
Supplement: S2 File — (PDF) [file pone.0175066.s002.pdf]

Table A. PERMANOVA for motile preservation experiment (OTU data).

	500 µm – 2mm		100 - 500 µm	
**Abundance Data**	**Pseudo-F**	**P**	**Pseudo-F**	**P**
ARMS	1.58	0.086	2.77	0.025
Preservation	1.1	0.432	1.27	0.228

Table B. PERMANOVA for motile preservation experiment, data merged by phylum.

	500 µm – 2mm		100 - 500 µm	
**Abundance Data**	**Pseudo-F**	**P**	**Pseudo-F**	**P**
ARMS	4.78	0.01	5.18	0.021
Preservation	2.49	0.156	3.24	0.055

Table C. ANOSIMs for motile preservation experiment (OTU data).

	500 µm – 2mm			100 - 500 µm		
**Abundance Data**	**Global R**	**P**	**Pairwise Tests**	**Global R**	**P**	**Pairwise Tests**
ARMS	0.753	0.004	No Differences	1	0.004	No Differences
Preservation	0	0.868	N/A	0.034	0.446	N/A

Table D. ANOSIMs for motile preservation experiment, data merged by phylum.

	500 µm – 2mm			100 - 500 µm		
**Abundance Data**	**Global R**	**P**	**Pairwise Tests**	**Global R**	**P**	**Pairwise Tests**
ARMS	0.547	0.014	No Differences	0.51	0.021	No Differences
Preservation	0.021	0.396	N/A	0.224	0.161	N/A
