# Supplementary material for: The importance of standardization for biodiversity comparisons: A case study using autonomous reef monitoring structures (ARMS) and metabarcoding to measure cryptic diversity on Mo’orea coral reefs, French Polynesia
Source: PLoS One. 2017 Apr 21;12(4):e0175066. doi: 10.1371/journal.pone.0175066 (PMC5400227; doi:10.1371/journal.pone.0175066)
Supplement: S3 File — (PDF) [file pone.0175066.s003.pdf]

Table A. One-Way ANOSIMs and Tukey Tests showing differences in community composition retrieved by processing method (OTU data). Sessile samples were split by preservation method (n=3 per treatment) prior to test. Tukey tests reported were calculated from abundance data.

	**EtOH**		**DMSO**		**RNAlater**		**Immediate Extraction**	
	**Global -R**	**p value**	**Global -R**	**p value**	**Global -R**	**p value**	**Global -R**	**p value**
ARMS	0.36	0.007	0.16	0.146	0.396	0.009	0.06	0.317
Tukey Tests significant differences	ARMS 1&2				ARMS 1&3			
Processing method	0.173	0.136	0.565	0.003	0.219	0.074	0.219	0.12
Tukey Tests significant differences			No significant diff					

Table B. One-Way OTU ANOSIMs and Tukey Tests showing differences in community composition retrieved by preservation method (OTU data). Sessile samples split by processing method (n=3 per treatment) prior to test. Tukey tests reported were calculated from abundance data.

	**NOAA**		**SWET**		**KEW**		**MILL**	
	**Global -R**	**p value**	**Global -R**	**p value**	**Global -R**	**p value**	**Global -R**	**p value**
ARMS	0.523	0.002	0.164	0.07	0.602	0.001	0.573	0.002
Tukey Tests significant differences	ARMS 1&2, 1&3				ARMS 1&2, 2&3, 1&3		ARMS 1&2	
Preservation method	0.33	0.027	0.312	0.007	0.253	0.063	-0.084	0.665
Tukey Tests significant differences	No significant diff		No significant diff					

Table C. One-Way ANOSIMs and Tukey Tests showing differences in community richness retrieved by processing and preservation method (OTU data). Sessile samples were split by processing method and preservation method (n=3 per treatment) prior to test. Tukey tests reported were calculated from richness data.

	**EtOH**		**DMSO**		**RNAlater**		**Immediate Extraction**	
	**Global -R**	**p value**	**Global -R**	**p value**	**Global -R**	**p value**	**Global -R**	**p value**
Processing method	0.2	0.131	0.403	0.011	0.093	0.267	0.42	0.022
Tukey Tests significant differences			No significant diff				No significant diff	
	**NOAA**		**SWET**		**KEW**		**MILL**	
	**Global -R**	**p value**	**Global -R**	**p value**	**Global -R**	**p value**	**Global -R**	**p value**
Preservation method	0.275	0.78	0.049	0.387	0.11	0.264	-0.019	0.901
Tukey Tests significant differences								
