# Supplementary material for: The importance of standardization for biodiversity comparisons: A case study using autonomous reef monitoring structures (ARMS) and metabarcoding to measure cryptic diversity on Mo’orea coral reefs, French Polynesia
Source: PLoS One. 2017 Apr 21;12(4):e0175066. doi: 10.1371/journal.pone.0175066 (PMC5400227; doi:10.1371/journal.pone.0175066)
Supplement: S4 File — (PDF) [file pone.0175066.s004.pdf]

Table A. One-Way ANOSIMs and Tukey Tests showing differences in community composition retrieved by processing method, data merged by phylum. Sessile samples were split by preservation method (n=3 per treatment) prior to test. Tukey tests reported were calculated from abundance data.

Processing Tests	EtOH		DMSO		RNAlater		Immediate Extraction	
	Global -R	p value	Global -R	p value	Global -R	p value	Global -R	p value
ARMS	0.107	0.21	-0.157	0.872	0.289	0.029	0.104	0.721
Tukey Tests significant differences								
Processing method	0.298	0.038	0.698	0.003	0.052	0.334	0.105	0.267
Tukey Tests significant differences	No significant diff		No significant diff					

Table B. One-Way ANOSIMs and Tukey Tests showing differences in community composition retrieved by preservation method, data merged by phylum. Sessile samples were split by processing method (n=3 per treatment) prior to test. Tukey tests reported were calculated from abundance data.

	NOAA		SWET		KEW		MILL	
	Global -R	p value	Global -R	p value	Global -R	p value	Global -R	p value
ARMS	0.069	0.279	0.123	0.138	0.218	0.055	0.391	0.011
Tukey Tests significant differences							No significant diff	
Preservation method	0.528	0.003	0.364	0.003	0.281	0.034	0.111	0.257
Tukey Tests significant differences	No significant diff		No significant diff		No significant diff			
