# Supplementary material for: The importance of standardization for biodiversity comparisons: A case study using autonomous reef monitoring structures (ARMS) and metabarcoding to measure cryptic diversity on Mo’orea coral reefs, French Polynesia
Source: PLoS One. 2017 Apr 21;12(4):e0175066. doi: 10.1371/journal.pone.0175066 (PMC5400227; doi:10.1371/journal.pone.0175066)
Supplement: S5 File — (PDF) [file pone.0175066.s005.pdf]

Table A. SIMPER analysis showing Phylum level differences in community composition across all data from the sessile processing experiment, as retrieved by processing method (top table) and preservation technique (bottom table). Values to the left of the tables show within group similarities for comparison with between group average differences. Percentage contribution of the most important phyla to community differences are reported with brackets to indicate which method returned the higher value for each phyla (e.g. (N) for NOAA). Most important differences and phyla contributions are reported in bold; highlighted cells indicate patterns in data.

All Abundance Data	Method	Similarity within Group	Compare Method	Ave Diff	Rhodophyta	Annelida	Cnidaria	Bryzoan	Porifera	Mollusca	Unknown
Processing Method			NOAA/ SWET	21.39	**18.88 (N)**	9.97 (S)	11 (S)		12.47 (S)		9.28 (N)
	NOAA	81.98	NOAA/ KEW	**24.34**	**20.31 (N)**	16.31 (K)	10.92 (K)		8.26 (K)		7.81 (N)
	SWET	81.42	NOAA/ MILL	**27.06**	**18.31 (N)**	11.15 (M)		10.58 (M)		9.91 (M)	9.64 (N)
	KEW	79.14	SWET/ KEW	19.77	**16.82 (S)**	14.77 (K)	9.85 (K)	8.92 (S)	13.66 (S)		
	MILL	79.58	SWET/ MILL	22.11	**14.83 (S)**	10.72 (M)		12.21 (M)	12.31 (S)	11.95 (M)	
			KEW/ MILL	21.95	13.3 (K)	**13.5 (K)**		13.04 (M)	11.09 (M)	12.21 (M)	

All Abundance Data	Method	Similarity within Group	Compare Method	Ave Diff	Rhodophyta	Annelida	Cnidaria	Bryzoa	Porifera	Mollusca	Unknown
Preservation Technique			EtOH/ DMSO	21.94	**15.37 (D)**	12.77 (D)	9.97 (E)	9.78 (D)	13.99 (E)		
	EtOH	79.53	EtOH/ RNAlater	**24.03**	**19.76 (RL)**	9.8 (RL)	10.06 (E)	8.12 (E)	14.58 (E)		
	DMSO	78.87	EtOH/ IMM	22	11.74 (E)	**12.96 (I)**	11.85 (E)	10.28 (I)	11.5 (E)		
	RNAlater	79.7	DMSO/ RNAlater	21.79	**18.29 (RL)**	14.65 (D)		11.51 (D)	7.03 (D)		9.12 (RL)
	IMM	78.58	DMSO/ IMM	22.82	**16.24 (D)**	13.65 (I)		11.33 (I)	8.81 (I)	7.91 (I)	
			RNAlater/ IMM	**26.39**	**22.9 (RL)**	12.17 (I)	6.99 (RL)	10.71 (I)	8.48 (I)		

Table B. SIMPER analysis showing phylum level differences in community composition due to processing method and partitioned by preservation method (top two tables) and differences in community composition due to preservation method and partitioned by processing method (bottom two tables) from the sessile processing experiment, to provide more detail of the important phyla. Red text represents subsets of data containing significant differences as calculated by ANOSIM; green cells represent areas where we see decrease/increase in importance of certain phyla with certain processing/preservation methods (n=3 per treatment; abundance data). Most important differences and phyla contributions are reported in bold; highlighted cells indicate patterns in data.

**Subset of Abundance Data**	**Method**	**Similarity within Group**	**Compare Method**	**Ave Diff**	**Rhododphyta**	**Annelida**	**Cnidaria**	**Bryzoan**	**Porifera**	**Mollusca**	**Unknown**	**Heterokontophyta**
			NOAA/SWET	22	**16.41 (N)**		13.79 (S)	8.53 (N)	14.89 (S)		8.99 (N)	
	NOAA	83.9	NOAA/KEW	22	**16.39 (N)**		16.36 (K)	12.02 (N)	10.18 (K)			7.47 (N)
**EtOH Stored Samples**	SWET	82.5	NOAA/MILL	23.2	13.6 (N)				**16.2 (M)**	11.6 (M)	8.20 (N)	8.02 (N)
	KEW	80.6	SWET/KEW	15.9	12.64 (S)	12.23 (K)	**18.94 (K)**	9.37 (S)	11.22 (S)			
	MILL	74.6	SWET/MILL	21.1	13.05 (M)	7.55 (M)	13.45 (S)		**18.9 (S)**	12.09 (M)		
			KEW/MILL	22.8	14.00 (M)		15.29 (K)	9.71 (M)	**17.01 (K)**	12.56 (M)		
			NOAA/SWET	21.5	**21.63 (N)**	11.70 (S)	8.21 (S)	9.11 (S)	12.32 (S)			
	NOAA	85.8	NOAA /KEW	**27.2**	**27.31 (N)**	21.72 (K)		9.45 (K)	4.80 (K)			7.34 (N)
**DMSO Stored Samples**	SWET	87.5	NOAA/MILL	**31.1**	**23.8 (N)**	9.3 (M)		15.3 (M)		8.91 (M)		8.00 (N)
	KEW	81.1	SWET/KEW	18.3	14.07 (S)	**21.41 (K)**		10.90 (K)	10.45 (S)			
	MILL	86.5	SWET/MILL	18.4	13.83 (S)	11.06 (M)		**14.65 (M)**	8.56 (S)	14.5 (M)		
			KEW/MILL	18.7		**22.0 (K)**		14.04 (M)	7.68 (M)	14.93 (M)	10.46 (K)	

Subset of Abundance Data	Method	Similarity within Group	Compare Method	Ave Diff	Rhododphyta	Annelida	Cnidaria	Bryzoan	Porifera	Mollusca	Unknown	Heterokontophyta
			NOAA/ SWET	16.9	11.07 (N)	14.01 (S)	12.53 (S)				**16.05 (N)**	
	NOAA	80.8	NOAA/ KEW	21.9	12.86 (N)	**20.27 (K)**	14.88 (K)		5.29 (N)		13.43 (N)	
RNAlater Stored Samples	SWET	83.2	NOAA/ MILL	**26.7**	**17.3 (N)**	15.7 (M)	10.95 (M)			9.98 (M)	12.2 (N)	
	KEW	82.7	SWET/ KEW	16	11.70 (S)	**14.57 (K)**	8.36 (K)		8.75 (S)		8.47 (S)	
	MILL	74.1	SWET/ MILL	21.4	**16.97 (S)**	14.16 (M)		11.80 (M)		12.31 (M)	7.84 (S)	
			KEW/ MILL	19.6	**16.48 (K)**	13.45 (K)		12.60 (M)		12.79 (M)	9.06 (K)	
			NOAA/ SWET	21.5	12.89 (N)	**13.14 (S)**	7.92 (N)	10.68 (S)	8.47 (N)			
	NOAA	88.2	NOAA/ KEW	22.2	9.65 (N)	**10.00 (N)**	7.97 (N)	20.14 (K)				
Immediate extractions	SWET	73.8	NOAA/ MILL	23	12.6 (N)	**13.2 (M)**	8.58 (M)		8.24 (N)	11.7 (M)		
	KEW	79.2	SWET/ KEW	19.7		16.77 (S)		14.43 (K)	8.54 (S)	8.72 (S)	8.49 (S)	
	MILL	76.5	SWET/ MILL	20.8		**18.22 (M)**	9.00 (M)	9.60 (S)	12.21 (M)	13.38 (M)		
			KEW/ MILL	22.4		15.10 (M)	8.94 (M)	**18.04 (K)**	10.76 (M)	12.85 (M)		

**Subset of Abundance Data**	**Method**	**Similarity within Group**	**Compare Method**	**Ave Diff**	**Rhodophyta**	**Annelida**	**Cnidaria**	**Bryzoan**	**Porifera**	**Mollusca**	**Unknown**	**Arthropoda**	**Chordata**	**Heterokontophyta**
			EtOH/ DMSO	18.74	**23.46 (D)**	7.04 (E)	5.71 (E)	15.91 (E)	11.92 (E)					
	EtOH	83.86	EtOH/ RNAlater	20.88	**19.69 (RL)**		7.64 (E)	12.86 (E)	8.86 (E)		12.26 (RL)			
**NOAA Processed Samples**	DMSO	85.8	EtOH/ IMM	16.17	14.51 (E)	**16.32 (I)**	9.41 (I)	12.46 (E)	7.82 (I)					
	RNAlater	80.82	DMSO/ RNAlater	15.96	11.51 (D)	10.43 (D)	9.30 (D)				**17.58 (RL)**			9.15 (D)
	Immediate	88.17	DMSO/ IMM	**23.16**	**25.97 (D)**	12.89 (I)	6.93 (I)		13.30 (I)			6.10 (I)		
			RNAlater/ IMM	**25.2**	**22.40 (RL)**	16.22 (I)	8.67 (I)		9.08 (I)		9.77 (RL)			
			EtOH/ DMSO	17.37	**17.28 (D)**	12.18 (D)	13.88 (E)		15.76 (E)				6.03 (D)	
	EtOH	82.48	EtOH/ RNAlater	**24.31**	**25.73 (RL)**	7.28 (RL)	11.13 (E)		18.09 (E)		6.54 (RL)			
**SWET Processed Samples**	DMSO	87.47	EtOH/ IMM	22.78		**16.02 (I)**	14.82 (E)	10.21 (I)	14.13 (E)	7.50 (I)				
	RNAlater	83.24	DMSO/ RNAlater	17.02	**18.15 (RL)**	10.30 (D)		10.20 (D)	11.58 (D)		8.82 (RL)			
	Immediate	73.84	DMSO/ IMM	22.47	**19.31 (D)**	12.37 (I)	8.59 (D)	8.93 (I)	8.74 (D)					
			RNAlater/ IMM	**27.41**	**27.56 (RL)**	11.64 (I)		11.43 (I)	7.73 (I)			5.81 (I)		

Subset of Abundance Data	Method	Similarity within Group	Compare Method	Ave Diff	Rhododphyta	Annelida	Cnidaria	Bryzoan	Porifera	Mollusca	Unknown	Arthropoda	Chordata	Heterokontophyta
			EtOH/ DMSO	21.64	7.72 (D)	**22.95 (D)**	14.52 (E)	10.91 (D)	14.41 (E)					
	EtOH	80.55	EtOH/ RNAlater	**22.76**	**21.80 (RL)**	10.25 (RL)	10.48 (E)	6.46 (E)	19.65 (E)					
**KEW Processed Samples**	DMSO	81.07	EtOH/ IMM	**23.33**	8.72 (E)	9.55 (I)	18.02 (E)	**22.29 (I)**	9.52 (E)					
	RNAlater	82.67	DMSO/ RNAlater	20.47	**22.62 (RL)**	17.02 (D)		11.83 (D)	7.04 (D)		6.90 (RL)			
	Immediate	79.15	DMSO/ IMM	20.08		**22.20 (D)**	7.73 (D)	7.73 (I)	6.39 (I)		6.31 (D)			
			RNAlater/ IMM	**26.13**	**22.29 (RL)**	8.44 (RL)	8.83 (RL)	20.00 (I)	8.86 (I)					
			EtOH/ DMSO	19.02	14.09 (E)	8.45 (D)		11.20 (D)	**20.34 (E)**	10.70 (D)				
	EtOH	74.63	EtOH/ RNAlater	21.77	17.03 (RL)	13.76 (RL)		9.57 (E)	**17.66 (E)**	9.84 (E)				
MILL Processed Samples	DMSO	86.45	EtOH/ IMM	21.11	15.26 (E)	12.36 (I)	8.57 (I)		**18.40 (E)**	10.24 (I)				
	RNAlater	74.12	DMSO/ RNAlater	20.36	15.21 (RL)	14.99 (RL)		**18.68 (D)**	7.04 (D)	12.74 (D)				
	Immediate	76.46	DMSO/ IMM	22.06		**14.13 (I)**		13.78 (D)	10.97 (I)	12.71 (I)	10.34 (I)			
			RNAlater/ IMM	**25.41**	**19.09 (RL)**	15.52 (I)		8.79 (I)	10.47 (I)	10.97 (I)				
