# Supplementary material for: The importance of standardization for biodiversity comparisons: A case study using autonomous reef monitoring structures (ARMS) and metabarcoding to measure cryptic diversity on Mo’orea coral reefs, French Polynesia
Source: PLoS One. 2017 Apr 21;12(4):e0175066. doi: 10.1371/journal.pone.0175066 (PMC5400227; doi:10.1371/journal.pone.0175066)
Supplement: S6 File — (PDF) [file pone.0175066.s006.pdf]

Table A. One-Way OTU ANOSIMs and Tukey Tests showing differences in community composition retrieved by processing and preservation method, void of Rhodophyta (OTU data). Sessile samples split by preservation method (n=3 per treatment), prior to test. Tukey tests reported were calculated from abundance data.

	**EtOH**		**DMSO**		**RNAlater**		**Immediate Extraction**	
	**Global -R**	**p value**	**Global -R**	**p value**	**Global -R**	**p value**	**Global -R**	**p value**
Processing method<br>Tukey Tests significant differences	0.2	0.085	0.506<br>No significant diff	0.002	0.207	0.071	0.127	0.21
	**NOAA**		**SWET**		**KEW**		**MILL**	
	**Global -R**	**p value**	**Global -R**	**p value**	**Global -R**	**p value**	**Global -R**	**p value**
Preservation method<br>Tukey Tests significant differences	0.142	0.183	0.216	0.078	0.145	0.195	-0.098	0.704

Table B. One-Way ANOSIMs and Tukey Tests showing differences in community composition retrieved by processing and preservation method, data merged by phylum and void of Rhodophyta. Sessile samples were split by preservation and processing method (n=3 per treatment) prior to test. Tukey tests reported were calculated from abundance data.

	**EtOH**		**DMSO**		**RNAlater**		**Immediate Extraction**	
	**Global -R**	**p value**	**Global -R**	**p value**	**Global -R**	**p value**	**Global -R**	**p value**
Processing method<br>Tukey Tests significant differences	0.293<br>No significant diff	0.05	0.494<br>No significant diff	0.003	0.157	0.139	0.108	0.261
	**NOAA**		**SWET**		**KEW**		**MILL**	
	**Global -R**	**p value**	**Global -R**	**p value**	**Global -R**	**p value**	**Global -R**	**p value**
Preservation method<br>Tukey Tests significant differences	0.148	0.154	0.077	0.295	0.231	0.082	-0.12	0.775
