# Supplementary material for: The importance of standardization for biodiversity comparisons: A case study using autonomous reef monitoring structures (ARMS) and metabarcoding to measure cryptic diversity on Mo’orea coral reefs, French Polynesia
Source: PLoS One. 2017 Apr 21;12(4):e0175066. doi: 10.1371/journal.pone.0175066 (PMC5400227; doi:10.1371/journal.pone.0175066)
Supplement: S1 Table — Data shown represent phyla distribution among queries. (PDF) [file pone.0175066.s007.pdf]

S1 Table. Accuracy of relaxed parameters (≥85% sequence similarity) when assigning to higher-level taxonomic ranks with Blastn. Data shown represent phyla distribution among queries.

Phylum	Count	Match Phylum	%	Mismatch Phylum	Unidentified Phylum	%	Match Class	Mismatch Class	Match Order	Mismatch Order
Xenacoelomorpha	2	1	50	0	1	50	1	0	1	0
Porifera	5	5	100	0	0	0	4	1	2	3
Platyhelminthes	3	0	0	0	3	100	0	0	0	0
Nemertea	3	3	100	0	0	0	2	1	2	1
Mollusca	43	32	74	0	11	26	32	0	31	1
Hemichordata	2	0	0	0	2	100	0	0	0	0
Gastrotricha	1	0	0	0	1	100	0	0	0	0
Echinodermata	8	4	50	0	4	50	4	0	4	0
Ctenophora	1	0	0	0	1	100	0	0	0	0
Cnidaria	24	19	79	0	5	21	19	0	15	4
Chordata	14	13	93	0	1	7	13	0	13	0
Chaetognatha	1	1	100	0	0	0	1	0	1	0
Bryozoa	7	0	0	0	7	100	0	0	0	0
Brachiopoda	2	0	0	0	2	100	0	0	0	0
Arthropoda	88	60	68	0	28	32	58	2	58	2
Annelida	29	6	21	1	22	76	6	1	6	1
**Total**	**233**	**144**	**62**	**1**	**88**	**38**	**140**	**5**	**133**	**12**
