# Supplementary material for: The importance of standardization for biodiversity comparisons: A case study using autonomous reef monitoring structures (ARMS) and metabarcoding to measure cryptic diversity on Mo’orea coral reefs, French Polynesia
Source: PLoS One. 2017 Apr 21;12(4):e0175066. doi: 10.1371/journal.pone.0175066 (PMC5400227; doi:10.1371/journal.pone.0175066)
Supplement: S2 Table — (PDF) [file pone.0175066.s008.pdf]

S2 Table. Conflicting and altered taxonomies in database searches.

New phyla	Found in	Old phyla	Found in:	Note
Heterokontophyta	BOLD	Bacillariophyta	NCBI	
Rhodophyta	BOLD	Florideophyceae	NCBI	
Heterokontophyta	BOLD	Phaeophyceae	NCBI	
Sipuncula	BOLD, Biocode	Annelida	NCBI	when order is Sipuncula
Heterokontophyta	BOLD	Pinguiophyceae	NCBI	
Heterokontophyta	BOLD	Oomycetes	NCBI	
Sipuncula	BOLD	Annelida	NCBI	when order is Phascolosomatiformes
Pyrrophycophyta	BOLD	Dinophyceae	NCBI	
Rhodophyta	BOLD	Bangiophyceae	NCBI	
Rhodophyta	BOLD	Compsopogonophyceae	NCBI	
Heterokontophyta	BOLD	Haptophyceae	SAP	
Heterokontophyta	BOLD	Isochrysidales	NCBI	
Heterokontophyta	BOLD	Eustigmatophyceae	NCBI	
Amoebozoa	BOLD	Paramoebidae	NCBI	
Zygomycota	BOLD	Mucorales	NCBI	
Amoebozoa	BOLD	Squamamoeba	NCBI	
Apusozoa	BOLD	Apusomonadidae	NCBI	
Cercozoa	BOLD	Chlorarachnion	NCBI	

* BOLD nomenclature was favored over NCBI, for consistency.
