# Supplementary material for: The importance of standardization for biodiversity comparisons: A case study using autonomous reef monitoring structures (ARMS) and metabarcoding to measure cryptic diversity on Mo’orea coral reefs, French Polynesia
Source: PLoS One. 2017 Apr 21;12(4):e0175066. doi: 10.1371/journal.pone.0175066 (PMC5400227; doi:10.1371/journal.pone.0175066)
Supplement: S3 Table — Bold text represents most abundant phyla in each fraction. (PDF) [file pone.0175066.s009.pdf]

S3 Table. Phylum level identifications of OTUs and their relative abundance by ARMS fraction. Bold text represents most abundant phyla in each fraction.

	**106 - 500 µm Motile Fraction**		**500 µm - 2 mm Motile Fraction**		**Sessile Fraction**	
**Phylum**	OTUs	Relative abundance	OTUs	Relative abundance	OTUs	Relative abundance
Ambiguous	4	0.04	7	0.02	17	0.09
Amoebozoa	-	-	1	0.00	8	0.00
Annelida	**101**	**26.26**	**77**	**40.64**	144	11.96
Apusozoa	1	0.00	-	-	2	0.00
Arthropoda	**332**	**24.37**	**196**	**21.94**	447	2.89
Ascomycota	1	0.00	2	0.01	7	0.01
Basidiomycota	-	-	1	0.00	5	0.00
Bryozoa	45	2.17	38	1.76	**70**	**20.44**
Cercozoa	1	0.00	2	0.00	1	0.00
Chlorophyta	1	0.00	2	0.00	7	0.01
Chordata	9	0.06	6	0.08	23	0.44
Cnidaria	37	8.37	31	4.28	**63**	**16.17**
Echinodermata	11	0.39	13	0.75	15	0.04
Entoprocta	4	0.04	3	0.00	6	0.08
Gastrotricha	-	-	1	0.00	3	0.00
Hemichordata	1	0.02	-	-	1	0.02
Heterokontophyta	35	0.30	16	0.10	108	1.00
Mollusca	74	2.38	34	0.83	109	2.01
Myzostomida	-	-	-	-	1	0.00
Nematoda	5	0.01	1	0.00	17	0.03
Nemertea	8	0.06	5	0.08	9	0.02
Platyhelminthes	7	0.02	4	0.05	9	0.01
Porifera	19	5.75	**17**	**15.43**	**34**	**19.07**
Pyrrophycophyta	-	-	-	-	4	0.04
Rhodophyta	25	2.46	22	7.22	**54**	**16.60**
Rotifera	-	-	-	-	1	0.00
Sipuncula	5	0.51	8	0.19	12	0.39
Streptophyta	1	0.00	-	-	2	0.00
Tardigrada	-	-	-	-	2	0.00
Unidentified	**305**	**26.77**	139	6.61	1044	8.55
Xenacoelomorpha	-	-	-	-	1	0.00
Zygomycota	1	0.02	1	0.00	2	0.11
