# Supplementary material for: The importance of standardization for biodiversity comparisons: A case study using autonomous reef monitoring structures (ARMS) and metabarcoding to measure cryptic diversity on Mo’orea coral reefs, French Polynesia
Source: PLoS One. 2017 Apr 21;12(4):e0175066. doi: 10.1371/journal.pone.0175066 (PMC5400227; doi:10.1371/journal.pone.0175066)
Supplement: S4 Table — Bold values indicate low DNA quantity (<100 ng/ul) or quality (<20% >1000bp). Gel images were not available for immediately extracted samples. In the bottom table, values in bold indicate the processing and preservation methods with highest (bottom two rows) and lowest (top two rows) overall DNA quantity and quality. (PDF) [file pone.0175066.s010.pdf]

S4 Table. Raw data (top tables) and overall summary (bottom table) of the quantity of DNA and the percentage of that DNA that is > 1000 bp (as determined by ImageJ analysis) recovered from DNA extractions of sessile and motile ARMS samples across different processing and preservation methods. Bold values indicate low DNA quantity (<100 ng/ul) or quality (<20% >1000bp). Gel images were not available for immediately extracted samples. In the bottom table, values in bold indicate the processing and preservation methods with highest (bottom two rows) and lowest (top two rows) overall DNA quantity and quality.

ARMS	Sessile Processing Method	Preservation Method	DNA quantity (ng/ul)	Percentage of 'high quality' DNA (> 1000 bp) in sample
3	SWET	RNAlater	983	0.34
1	SWET	Immediate extraction	962	N/A
3	SWET	DMSO	910	0.38
2	SWET	Immediate extraction	602	N/A
1	SWET	DMSO	535	0.40
1	SWET	EtOH	449	**0.18**
3	SWET	Immediate extraction	342	N/A
2	SWET	DMSO	336	0.20
1	SWET	RNAlater	102	0.38
2	SWET	RNAlater	**97**	0.35
2	SWET	EtOH	**80**	**0.06**
3	SWET	EtOH	**67**	**0.09**

ARMS	Sessile Processing Method	Preservation Method	DNA quantity (ng/ul)	Percentage of 'high quality' DNA (> 1000 bp) in sample
1	MILL	DMSO	1900	0.45
2	MILL	DMSO	1000	**0.13**
2	MILL	Immediate extraction	436	N/A
1	MILL	Immediate extraction	193	N/A
3	MILL	DMSO	155	0.20
1	MILL	RNAlater	**77**	0.36
3	MILL	Immediate extraction	**68**	N/A
3	MILL	EtOH	**50**	0.33
2	MILL	RNAlater	**40**	0.30
3	MILL	RNAlater	**20**	0.24
2	MILL	EtOH	**19**	**0.18**

ARMS	Sessile Processing Method	Preservation Method	DNA quantity (ng/ul)	Percentage of 'high quality' DNA (> 1000 bp) in sample
1	NOAA	DMSO	273	0.60
2	NOAA	Immediate extraction	270	N/A
1	NOAA	EtOH	265	0.33
3	NOAA	DMSO	186	0.35
2	NOAA	DMSO	169	0.34
3	NOAA	Immediate extraction	154	N/A
1	NOAA	Immediate extraction	121	N/A
1	NOAA	RNAlater	100	0.40
3	NOAA	EtOH	**99**	**0.14**
2	NOAA	EtOH	**92**	0.24
3	NOAA	RNAlater	**50**	0.31
2	NOAA	RNAlater	**23**	0.37

ARMS	Sessile Processing Method	Preservation Method	DNA quantity (ng/ul)	Percentage of 'high quality' DNA (> 1000 bp) in sample
1	KEW	DMSO	2300	0.45
2	KEW	Immediate extraction	1400	N/A
1	KEW	Immediate extraction	1020	N/A
3	KEW	Immediate extraction	760	N/A
3	KEW	DMSO	665	**0.18**
2	KEW	DMSO	307	0.20
1	KEW	EtOH	140	**0.08**
1	KEW	RNAlater	138	0.33
3	KEW	RNAlater	132	0.20
2	KEW	RNAlater	100	0.33
2	KEW	EtOH	**88**	**0.06**
3	KEW	EtOH	**10**	0.23

ARMS	Motile Fraction	Preservation Method	DNA quantity (ng/ul)	Percentage of 'high quality' DNA (> 1000 bp) in sample
1	500 µm – 2 mm	DMSO	685	0.65
2	500 µm – 2 mm	DMSO	612	0.42
3	500 µm – 2 mm	DMSO	465	0.58
1	500 µm – 2 mm	EtOH	1350	0.47
2	500 µm – 2 mm	EtOH	606	0.60
3	500 µm – 2 mm	EtOH	1600	0.46
1	500 µm – 2 mm	RNAlater	1570	0.46
2	500 µm – 2 mm	RNAlater	548	0.60
3	500 µm – 2 mm	RNAlater	1560	0.45

ARMS	Motile Fraction	Preservation Method	DNA quantity (ng/ul)	Percentage of 'high quality' DNA (> 1000 bp) in sample
1	106 – 500 µm	DMSO	106	0.66
3	106 – 500 µm	DMSO	120	0.69
1	106 – 500 µm	EtOH	214	0.51
2	106 – 500 µm	EtOH	258	0.53
3	106 – 500 µm	EtOH	379	0.60
1	106 – 500 µm	RNAlater	158	0.42
2	106 – 500 µm	RNAlater	124	0.47
3	106 – 500 µm	RNAlater	222	0.41

**Summary by Processing and Preservation Method (% of samples)**	**SWET**	**NOAA**	**KEW**	**MILL**	**DMSO**	**EtOH**	**RNAlater**	**Immediate Extraction**
< 100 ng/ul DNA	25.0	33.3	16.7	**50.0**	0.0	**66.7**	50.0	8.3
> 80% low quality DNA (<1000bp)	**25.0**	8.3	**25.0**	16.7	16.7	**58.3**	0.0	N/A
20-40% high quality DNA (>1000bp)	41.7	**50.0**	41.7	41.7	50.0	33.3	**91.7**	N/A
40-60% high quality DNA (>1000bp)	8.3	**16.7**	8.3	8.3	**33.3**	0.0	8.3	N/A
