# Supplementary material for: The importance of standardization for biodiversity comparisons: A case study using autonomous reef monitoring structures (ARMS) and metabarcoding to measure cryptic diversity on Mo’orea coral reefs, French Polynesia
Source: PLoS One. 2017 Apr 21;12(4):e0175066. doi: 10.1371/journal.pone.0175066 (PMC5400227; doi:10.1371/journal.pone.0175066)
Supplement: S5 Table — (PDF) [file pone.0175066.s011.pdf]

S5 Table. PERMANOVA for sessile processing experiment (OTU data).

**Abundance Data**	**Pseudo-F**	**P**
ARMS	**5.03**	**0.001**
Processing	3.79	0.001
Preservation	3.12	0.001
ARMS x Processing	1.98	0.001
ARMS x Preservation	1.16	0.136
Processing x Preservation	1.27	0.006

**Abundance Data (void of immediately extracted samples)**	**Pseudo-F**	**P**
ARMS	**5.05**	**0.001**
Processing	3.77	0.001
Preservation	2.61	0.002
ARMS x Processing	1.95	0.001
ARMS x Preservation	1.39	0.04
Processing x Preservation	1.37	0.02

**Richness Data**	**Pseudo-F**	**P**
ARMS	**2.47**	**0.001**
Processing	2.05	0.002
Preservation	1.49	0.01
ARMS x Processing	1.32	0.006
ARMS x Preservation	1.08	0.253
Processing x Preservation	1.08	0.156
Processing (void of immediately extracted samples)	1.78	0.002
