# Supplementary material for: The importance of standardization for biodiversity comparisons: A case study using autonomous reef monitoring structures (ARMS) and metabarcoding to measure cryptic diversity on Mo’orea coral reefs, French Polynesia
Source: PLoS One. 2017 Apr 21;12(4):e0175066. doi: 10.1371/journal.pone.0175066 (PMC5400227; doi:10.1371/journal.pone.0175066)
Supplement: S7 Table — Based on rarefied abundance data, void of singletons. (PDF) [file pone.0175066.s013.pdf]

S7 Table. Diversity data for sessile processing experiment, showing number of OTUs (S), species richness (Margalef; d), Pielou's eveness (J'), and Shannon diversity (H'). Based on rarefied abundance data (n=5899), void of singletons.

ARMS	Processing Method	Preservation Method	S	d	J'	H' (log_e)
1	SWET	EtOH	275	31.56	0.606	3.404
2	SWET	EtOH	183	20.96	0.5561	2.897
3	SWET	EtOH	175	20.04	0.5164	2.667
1	SWET	DMSO	260	29.83	0.5716	3.179
2	SWET	DMSO	253	29.02	0.5831	3.227
3	SWET	DMSO	308	35.36	0.5659	3.242
1	SWET	RNAlater	211	24.19	0.5935	3.176
2	SWET	RNAlater	226	25.91	0.6135	3.326
3	SWET	RNAlater	238	27.3	0.635	3.475
1	SWET	Immediate Extract	210	24.07	0.542	2.898
2	SWET	Immediate Extract	327	37.55	0.658	3.81
3	SWET	Immediate Extract	168	19.23	0.5274	2.702
1	KEW	EtOH	257	29.48	0.6072	3.369
2	KEW	EtOH	196	22.46	0.6042	3.189
3	KEW	EtOH	162	18.54	0.4456	2.267
1	KEW	DMSO	200	22.92	0.528	2.798
2	KEW	DMSO	162	18.54	0.5914	3.009
3	KEW	DMSO	255	29.25	0.5215	2.89
1	KEW	RNAlater	215	24.65	0.555	2.981
2	KEW	RNAlater	234	26.84	0.5849	3.191
3	KEW	RNAlater	243	27.87	0.5945	3.265
1	KEW	Immediate Extract	208	23.84	0.5272	2.814
2	KEW	Immediate Extract	188	21.54	0.4759	2.492
3	KEW	Immediate Extract	344	39.5	0.6705	3.916
2	MILL	EtOH	169	19.35	0.5543	2.844
3	MILL	EtOH	101	11.52	0.6101	2.816
1	MILL	DMSO	211	24.19	0.4969	2.66
2	MILL	DMSO	200	22.92	0.5526	2.928
3	MILL	DMSO	143	16.35	0.5362	2.661
1	MILL	RNAlater	210	24.07	0.5177	2.768
2	MILL	RNAlater	164	18.77	0.5465	2.787
3	MILL	RNAlater	183	20.96	0.5814	3.029
1	MILL	Immediate Extract	205	23.5	0.5009	2.666
2	MILL	Immediate Extract	226	25.91	0.5642	3.058
3	MILL	Immediate Extract	140	16.01	0.5627	2.781
1	NOAA	EtOH	420	48.26	0.6266	3.785
2	NOAA	EtOH	239	27.41	0.6414	3.513
3	NOAA	EtOH	280	32.13	0.6236	3.514
1	NOAA	DMSO	256	29.37	0.5405	2.997
2	NOAA	DMSO	277	31.79	0.6108	3.435
3	NOAA	DMSO	331	38.01	0.6193	3.594
1	NOAA	RNAlater	232	26.61	0.5151	2.806
2	NOAA	RNAlater	212	24.3	0.6137	3.287
3	NOAA	RNAlater	246	28.22	0.6737	3.709
1	NOAA	Immediate Extract	388	44.57	0.6097	3.634
2	NOAA	Immediate Extract	352	40.43	0.6131	3.595
3	NOAA	Immediate Extract	437	50.22	0.6468	3.932
