# Supplementary material for: The importance of standardization for biodiversity comparisons: A case study using autonomous reef monitoring structures (ARMS) and metabarcoding to measure cryptic diversity on Mo’orea coral reefs, French Polynesia
Source: PLoS One. 2017 Apr 21;12(4):e0175066. doi: 10.1371/journal.pone.0175066 (PMC5400227; doi:10.1371/journal.pone.0175066)
Supplement: S8 Table — (PDF) [file pone.0175066.s014.pdf]

S8 Table. PERMANOVA for sessile processing experiment, data merged by phylum.

**Abundance Data**	**Pseudo-F**	**P**
ARMS	6.36	0.001
Processing	6.64	0.001
Preservation	**7.33**	**0.001**
ARMS x Processing	2.8	0.001
ARMS x Preservation	1.26	0.232
Processing x Preservation	2.01	0.003

**Abundance Data (void of immediately extracted samples)**	**Pseudo-F**	**P**
ARMS	7.25	0.001
Processing	**9.41**	**0.001**
Preservation	7.97	0.001
ARMS x Processing	3.04	0.001
ARMS x Preservation	2.07	0.045
Processing x Preservation	2.22	0.01

**Richness Data**	**Pseudo-F**	**P**
ARMS	5.43	0.001
Processing	**6.53**	**0.001**
Preservation	4.47	0.001
ARMS x Processing	1.68	0.029
ARMS x Preservation	1.45	0.097
Processing x Preservation	1.31	0.124
Processing (void of immediately extracted samples)	**4.98**	**0.001**
