# Supplementary material for: The importance of standardization for biodiversity comparisons: A case study using autonomous reef monitoring structures (ARMS) and metabarcoding to measure cryptic diversity on Mo’orea coral reefs, French Polynesia
Source: PLoS One. 2017 Apr 21;12(4):e0175066. doi: 10.1371/journal.pone.0175066 (PMC5400227; doi:10.1371/journal.pone.0175066)
Supplement: S9 Table — ANOSIMs were carried out across all data for ARMS, processing method and preservation method. Tukey tests reported were calculated from all abundance data (* p ≤ 0.005, ** p ≤ 0.05, *** p > 0.05). (PDF) [file pone.0175066.s015.pdf]

S9 Table. ANOSIMs and Tukey Tests for sessile processing experiment, data merged by phylum. ANOSIMs were carried out across all data for ARMS, processing method and preservation method. Tukey tests reported were calculated from all abundance data (* p ≤ 0.005, ** p ≤ 0.05, *** p > 0.05).

	**Abundance Global-R**	**Richness Global-R**
ARMS	0.106**	0.132*
Processing	0.198*	**0.290***
Preservation	**0.206***	0.156*
Processing (void of immediately extracted samples)	**0.299***	**0.290***

**ARMS Tukey Tests**	**ARMS 1 R value**	**ARMS 2 R value**
ARMS 1		
ARMS 2	**0.185***	
ARMS 3	0.05***	0.092**

**Processing Tukey Tests**	**NOAA R value**	**SWET R value**	**KEW R value**
NOAA			
SWET	0.179**		
KEW	0.326*	0***	
MILL	**0.549***	0.113**	0.035***

**Preservation Tukey Tests**	**EtOH R value**	**DMSO R value**	**RNAlater R value**
EtOH			
DMSO	0.116**		
RNAlater	0.33*	0.087***	
Immediately Extracted	0.084***	0.126**	0.487*
