# Supplementary material for: The importance of standardization for biodiversity comparisons: A case study using autonomous reef monitoring structures (ARMS) and metabarcoding to measure cryptic diversity on Mo’orea coral reefs, French Polynesia
Source: PLoS One. 2017 Apr 21;12(4):e0175066. doi: 10.1371/journal.pone.0175066 (PMC5400227; doi:10.1371/journal.pone.0175066)
Supplement: S10 Table — Tables show phylum level differences in community composition between SIMPROF clusters, which compared community composition data retrieved from different processing methods and CPCe image analysis, for all three ARMS (Fig 5). * represents the cluster containing the community as determined by CPCe image analysis. Values to the left of the tables show within group similarities for comparison with between group average differences. Percentage contributions of the most important phyla to community differences are reported with brackets to indicate which cluster returned the higher value for each phyla. (PDF) [file pone.0175066.s016.pdf]

S10 Table. SIMPER analysis of the subset of the communities retrieved from the sessile processing experiment, used in comparison with CPCe (Coral Point Count) data. Table shows phylum level differences in community composition between SIMPROF clusters, which compared community composition data retrieved from different processing methods and CPCe image analysis, for all three ARMS (Fig 5). * represents the cluster containing the community as determined by CPCe image analysis. Values to the left of the tables show within group similarities for comparison with between group average differences. Percentage contribution of the most important phyla to community differences are reported with brackets to indicate which cluster returned the higher value for each phyla.

**ARMS 1**	**Similarity within Group**	**Cluster Comparison**	**Average Difference**	**Rhodophyta**	**Porifera**	**Bryozoa**
**Cluster 1***	87.91	**1* & 2**	20.12	35.05 (**1***)	35.09 (2)	9.47 (2)
Cluster 2	94.06	**1* & 3**	25.62	40.93 (**1***)	9.55 (3)	31.79 (3)
Cluster 3	88.4	**2 & 3**	19.93	19.19 (2)	25.40 (2)	33.69 (3)

**ARMS 2**	**Similarity within Group**	**Cluster Comparison**	**Average Difference**	**Rhodophyta**	**Porifera**	**Bryozoa**	**Bivalvia**
Cluster 1	90.62	**1 & 2***	18.44	35.93 (1)	20.73 (**2***)	19.44 (**2***)	
**Cluster 2***	88.52	**1 & 3**	31.67	41.22 (1)	15.30 (3)		16.25 (3)
Cluster 3	81.08	**2* & 3**	20.19	31.33 (**2***)	16.61 (3)		24.19 (3)

**ARMS 3**	**Similarity within Group**	**Cluster Comparison**	**Average Difference**	**Rhodophyta**	**Porifera**	**Bryozoa**
**Cluster 1***	83.82	**1* & 2**	30.77	39.87 (**1***)	26.01 (2)	18.10 (2)
Cluster 2	84.89					
